# Unveiling the axonal connectivity between the precuneus and temporal pole: Structural evidence from the cingulum pathways

**DOI:** 10.1002/hbm.26771

**Published:** 2024-06-25

**Authors:** Georgios P. Skandalakis, Wen‐Jieh Linn, Fang‐Cheng Yeh, Syed Faraz Kazim, Spyridon Komaitis, Eleftherios Neromyliotis, Dimitrios Dimopoulos, Evangelos Drosos, Constantinos G. Hadjipanayis, Paul N. Kongkham, Gelareh Zadeh, George Stranjalis, Christos Koutsarnakis, Michael Kogan, Linton T. Evans, Aristotelis Kalyvas

**Affiliations:** ^1^ Section of Neurosurgery Dartmouth Hitchcock Medical Center Lebanon New Hampshire USA; ^2^ Department of Neurosurgery National and Kapodistrian University of Athens School of Medicine Athens Greece; ^3^ Department of Neurological Surgery University of Pittsburgh Pittsburgh Pennsylvania USA; ^4^ Department of Neurosurgery University of New Mexico Hospital Albuquerque New Mexico USA; ^5^ Department of Neurosurgery Toronto Western Hospital, University Health Network Toronto Ontario Canada

**Keywords:** cingulum, parahippocampus, precuneus, temporal pole, tractography

## Abstract

Neuroimaging studies have consistently demonstrated concurrent activation of the human precuneus and temporal pole (TP), both during resting‐state conditions and various higher‐order cognitive functions. However, the precise underlying structural connectivity between these brain regions remains uncertain despite significant advancements in neuroscience research. In this study, we investigated the connectivity of the precuneus and TP by employing parcellation‐based fiber micro‐dissections in human brains and fiber tractography techniques in a sample of 1065 human subjects and a sample of 41 rhesus macaques. Our results demonstrate the connectivity between the posterior precuneus area POS2 and the areas 35, 36, and TG of the TP via the fifth subcomponent of the cingulum (CB‐V) also known as parahippocampal cingulum. This finding contributes to our understanding of the connections within the posteromedial cortices, facilitating a more comprehensive integration of anatomy and function in both normal and pathological brain processes.

**Practitioner Points:**

Our investigation delves into the intricate architecture and connectivity patterns of subregions within the precuneus and temporal pole, filling a crucial gap in our knowledge.

- We revealed a direct axonal connection between the posterior precuneus (POS2) and specific areas (35, 35, and TG) of the temporal pole.
- The direct connections are part of the CB‐V pathway and exhibit a significant association with the cingulum, SRF, forceps major, and ILF.
- Population‐based human tractography and rhesus macaque fiber tractography showed consistent results that support micro‐dissection outcomes.

## Graphical abstract

The study investigated the connectivity of the precuneus and temporal pole by employing parcellation‐based fiber micro‐dissections in human brains and fiber tractography techniques in a sample of 1065 human subjects and a sample of 41 rhesus macaques. The results demonstrate the connectivity between the posterior precuneus area POS2 and the areas 35, 36, TG, and TI of the temporal pole via the fifth subcomponent of the cingulum (CB‐V) also known as parahippocampal cingulum.

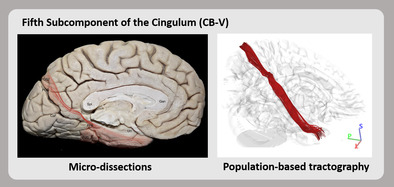

## 1 INTRODUCTION

The functional connectivity between the precuneus and temporal pole (TP) has garnered considerable attention in recent literature, establishing their vital roles within the default mode network and cognitive processes (Alves et al., 2019; Aubinet et al., 2018; Pascual et al., 2015; Rolls, Deco, et al., 2022; Rolls, Wirth, et al., 2022). These regions exhibit synchronous activation during resting‐state conditions and play crucial roles in verbal creativity, episodic memory, emotional regulation, and social cognition in healthy individuals (Adnan et al., 2016; Pehrs et al., 2017; Sun et al., 2018; Wade‐Bohleber et al., 2018). Conversely, disruptions in this coordinated activation have been observed in various neuropsychiatric conditions, underscoring the clinical significance of understanding their underlying structural connectivity (Cao et al., 2022; Fuentes‐Claramonte et al., 2022; Yin et al., 2022).

The structural connectivity between the precuneus and TP has been demonstrated by the MdLF (Kalyvas et al., 2020; Sasaki et al., 2023). The MdLF is a fiber tract interconnecting the precuneus with areas TA, and TG of the TP (Kalyvas et al., 2020). Nevertheless, the TP is composed by additional functionally distinctive regions (Ding et al., 2009) and their structural connectivity with the precuneus and its underlying subregions is not well understood (Luo et al., 2020; Tanglay et al., 2022). Specifically, the connectivity among regions 35, 36, and TI with the precuneus has not been demonstrated (Jung et al., 2017; Sasaki et al., 2023; Vos de Wael et al., 2021). Given the wide array of functions implicated by the synchronous activation of the precuneus and TP, we hypothesize that these functions are subserved by a complex structural circuitry involving more than a single fiber tract that is the MdLF. Therefore, we also hypothesize that areas 35,36, and TI are connected with the precuneus via a distinct fiber tract.

To further elucidate the organization of these regions, we performed parcellation based micro‐dissection guided fiber tractography in human and rhesus macaques. We utilized human cadaveric hemispheres and high‐resolution diffusion‐weighted imaging (DWI) datasets from 1115 subjects ensuring a robust analysis. Here, we show the direct axonal connectivity of area POS2 of the precuneus with areas 35,35, and TG of the TP through a distinct long association fiber tract exhibiting the characteristics of the 5th component of the cingulum. Our results refine the current knowledge on structural brain connectivity and organization in terms of parcellation‐based axonal interactions. Findings of this study could facilitate research of pathophysiological mechanisms underlying various neuropsychiatric conditions. Ultimately, unraveling the complex connectivity between the precuneus and TP will contribute to the broader understanding of brain architecture and facilitate the development of targeted interventions for neurological and psychiatric disorders.

## 2 METHODS

### 2.1 Micro‐dissections

Nine (9) normal, adult, cerebral hemispheres were obtained from nine different individuals in routine autopsy and were fixed in a 10% formalin solution for 8 weeks. Following the dissection of the arachnoid membrane and vessels, all cerebral hemispheres were prepared according to the Klingler's technique (Wang et al., 2012; Zemmoura et al., 2016) and were subsequently investigated through the fiber micro‐dissection technique and the surgical microscopes (Carl Zeiss OPMIR Plus, Carl Zeiss AG, Oberkochen, Germany) as previously described (Kalyvas et al., 2020; Komaitis et al., 2022; Skandalakis et al., 2020; Skandalakis et al., 2024). Digital photographs were obtained in different dissection levels and angles to adequately illustrate the structural and topographical architecture of this newly identified connection. Fiber micro‐dissections were independently carried out by three different authors (G.P.S., S.K., A.K.).

In this study, we focused our attention on the connectivity of the precuneus and TP according to their parcellation. Fibers terminating to the precuneus were confined in the anterior bank of the POS (parietooccipital sulcus) corresponding to the area POS2 of the HCP MMP1.0 atlas (Glasser, Coalson, et al., 2016a; Glasser, Smith, et al., 2016b). To study the termination points within the different regions of the temporopolar cortex, we studied the areas 35,36, TI, and TG as described by Ding et al. (2009).

We initiated the micro‐dissection process by identifying regional cortical and sulcal points. Specifically, we resected the cortex and underlying short association fibers, also known as arcuate or “U” fibers, starting from the parieto‐occipital sulcus and extending toward the distal end of the calcarine sulcus. The micro‐dissection process involved a gradual progression around the parieto‐occipital sulcus and calcarine fissure, including the cuneus, precuneus, posterior cingulate cortex, retrosplenial cortex, ventral and medial temporal lobe, and finally reaching the anterior region encompassing the TP. Fiber micro‐dissections allowed us to assess the tracts connecting the precuneus and TP, documenting their topography, morphology, termination points, and their relationship with other subcortical pathways.

The study received approval from the ethical board at the University of Athens (01.14.2019/protocol number: 067), and all procedures performed adhered to the ethical standards of the institutional and/or national research committee, as well as the 1964 Helsinki Declaration and its later amendments or comparable ethical standards.

### 2.2 Human tractography

The tract was reconstructed bilaterally in a built‐in population‐average human template and 40 hemispheres from 20 individual healthy adult subjects from the HCP. DSI Studio was used for reconstruction of the tracts (Yeh et al., 2022). The template was generated from imaging data of 1065 subjects acquired from the Human Connectome Project (Glasser et al., 2016b) (WashU consortium) using DSI Studio software. The diffusion data were acquired by *b*‐values of 1000, 2000, and 3000 s/mm^2^. The number of diffusion sampling directions were 90, 90, and 90, respectively. The in‐plane resolution was 1.25 mm. The slice thickness was 1.25 mm. The *b*‐table was checked by an automatic quality control routine to ensure its accuracy (Schilling et al., 2019). The diffusion data were reconstructed in the MNI space using *q*‐space diffeomorphic reconstruction (Yeh & Tseng, 2011) to obtain the spin distribution function (Yeh et al., 2010). A diffusion sampling length ratio of 1.7 was used. The restricted diffusion was quantified using restricted diffusion imaging (Yeh et al., 2017). A two regions of interest (ROI) approach used for fiber tracking. ROIs were created according to the extended HCP atlas (Huang et al., 2022). First ROI was POS2 and second ROI included the combination of TGv, TGd, areas 35 and 36. Length and volume were measured bilaterally on reconstructions of the tract on the population‐based averaged template. Length, volume, and fractional anisotropy were measured using subject‐specific tractography in 40 hemispheres. To assess for potential lateralization of the tract, volumes between the left and right hemispheres were compared using the volume lateralization index $\mathrm{LIv}=\frac{\left(\mathrm{Lv}-\mathrm{Rv}\right)}{\left(\mathrm{Lv}+\mathrm{Rv}\right)}$ as previously described (Catani et al., 2007; Howells et al., 2018). Mean ± SD of length, volume, fractional anisotropy, and volume lateralization index of the tracts were also analyzed and reported.

To illustrate the relationship between the CB‐V and adjacent structures, we reconstructed the, ILF, MdLF, forceps major, and tapetum fibers as previously reported (Fernandez‐Miranda et al., 2012; Kalyvas et al., 2020; Panesar et al., 2018; Skandalakis et al., 2023) using the 1065 HCP averaged template. Moreover, we overlayed the tract on a 100 μm resolution MR dataset (Edlow et al., 2019) which provides histologic like analysis.

### 2.3 Qualitative connectivity analysis and connectogram creation

To assess the subregions of the TP and precuneus implicated in the connectivity of the fiber tract under investigation, we qualitatively assessed the reconstructed fiber tracts under visual inspection of the fiber tracts in 3D in multiple views. This evaluation was conducted using DSI studio, wherein the detailed structure of the tract was rotated in all directions, followed on a “slice‐by‐slice” basis, and assessed in 3D space relative to adjacent structures such as cortical structures, white matter tracts, and sulci along the route of the dMRI streamlines. A connectogram was generated from single‐subject qualitative connectivity data using the CIRCOS data visualization tool (http://mkweb.bcgsc.ca/tableviewer/visualize/).

### 2.4 Rhesus macaque tractography

The built‐in population‐average rhesus monkey template in DSI Studio was used to obtain the rhesus macaque tractography. The template was constructed from a dataset comprising 41 scans from PRIMatE Data Exchange (PRIME‐DE) (Milham et al., 2020). The MRI data were acquired using a multishell diffusion scheme with *b*‐values of 1250 and 2500 s/mm^2^. Imaging parameters included an in‐plane resolution and slice thickness of 0.5 mm. To address susceptibility artifacts, reversed phase‐encoding b0 by TOPUP from the Tiny FSL package (http://github.com/frankyeh/TinyFSL), a re‐compiled version of FSL TOPUP (FMRIB, Oxford) with multi‐thread support, was employed. Eddy current distortion was corrected using FSL eddy within the integrated interface of DSI Studio (“Chen” release) (http://dsi-studio.labsolver.org). The accuracy of the *b*‐table orientation was assessed by comparing fiber orientations with those of a population‐averaged template (Yeh et al., 2018). The diffusion data was reconstructed in the MNI space using *q*‐space diffeomorphic reconstruction (Yeh & Tseng, 2011) to derive the spin distribution function (Yeh et al., 2010). Diffeomorphic reconstruction utilized a diffusion sampling length ratio of 1.25, and the resulting output resolution was 1.0 mm isotropic.

For fiber tracking, a deterministic fiber tracking algorithm (Yeh et al., 2013) was utilized, incorporating augmented tracking strategies (Yeh, 2020) to enhance reproducibility. Automatic fiber tracking selecting parahippocampal cingulum as a target (Cingulum_Parahippocampal_Parietal_L, Cingulum_Parahippocampal_Parietal_R) was performed to reconstruct tracts interconnecting the precuneus and TP through the cingulum pathways. To allow for variability, the anisotropy threshold and angular threshold were randomly selected within ranges of 15 degrees to 90 degrees. Additionally, the step size was randomly chosen from 0.5 voxel to 1.5 voxels. Tracks with lengths shorter than 7 mm or exceeding 160 mm were discarded to ensure data quality. Ultimately, a total of 10,000 tracts were calculated, enabling comprehensive analysis and interpretation of the diffusion data.

## 3 RESULTS

### 3.1 Micro‐dissection

**FIGURE 1:**
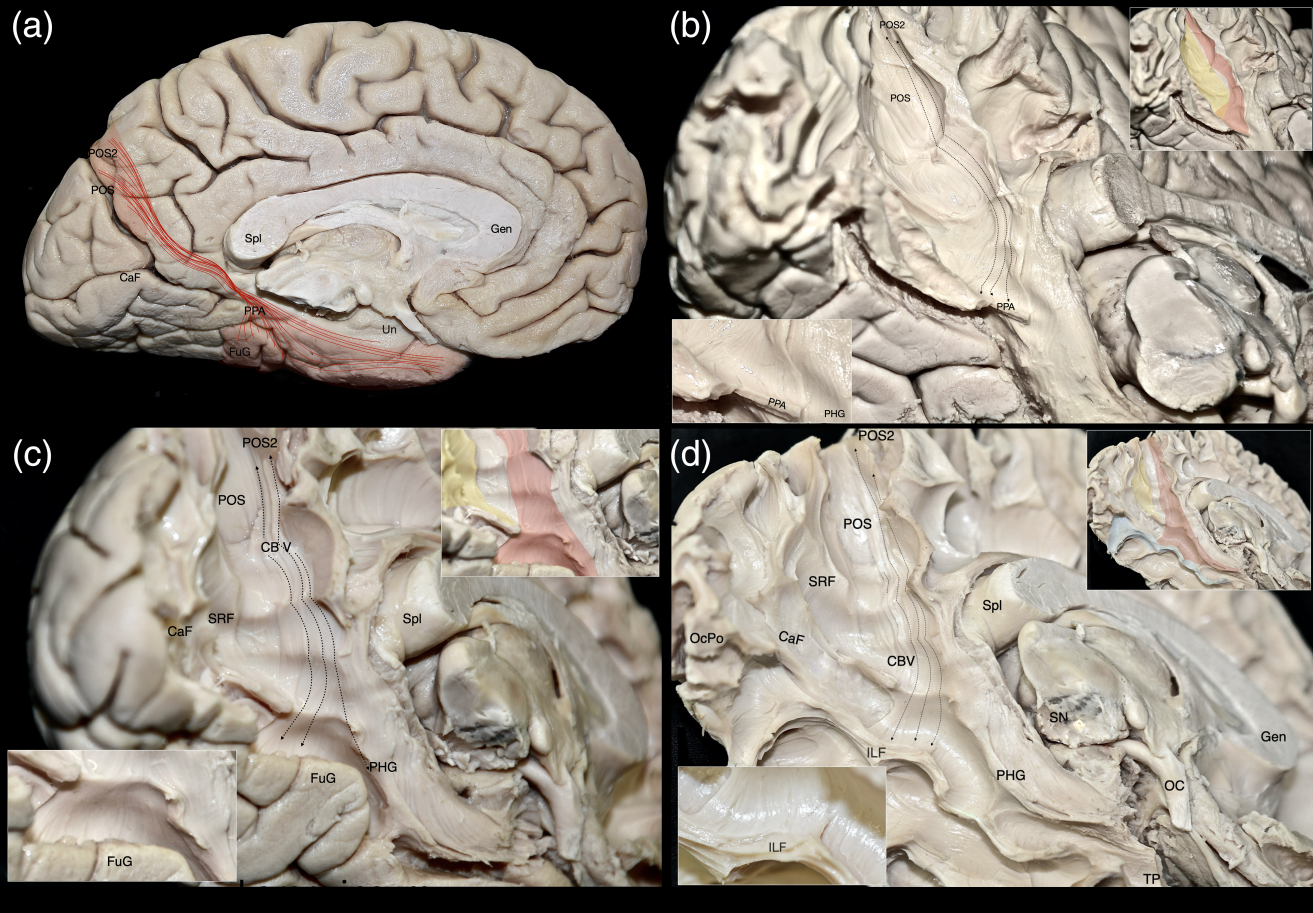
Micro‐dissection representations on a stepwise manner. (a) Representation of trajectory and cortical projections of the CB‐V superimposed on the medial aspect of a left hemisphere. (b) Illustration of the CB‐V fibers interconnecting the POS and PPA. Left hemisphere, infero‐medial view depicting the regional white matter anatomy following the micro‐dissection of the cortex and underlying U‐fibers of the cuneus precuneus, retrosplenial cortex, and lingual gyrus. CB‐V fibers running between the POS2 and PPA can be observed at the anterior bank of the POS. The SRF can be observed traveling in an oblique rostro‐ventral direction from the rostral cuneus (posterior bank of POS) toward the PPA. Upper inset: Inset: The CB‐V is highlighted in red and SRF in yellow. Lower inset: Close‐up of the inferior terminations of the CB‐V in the PPA. The trajectory of the CB‐V is outlined with black dotted arrows. (c) Illustration of the CB‐V fibers interconnecting the POS2, fusiform, and parahippocampal gyrus. The left hemisphere, infero‐medial view depicting the regional white matter anatomy following resection of SRF and CB‐V fibers terminating at the PPA. Longer CB‐V fibers terminating in the anterior parahippocampal gyrus and fusiform gyrus are observed. Notice the lateral curve and widening they display below the level of the PPA. Upper inset: Inset: The CB‐V is highlighted in red and SRF in yellow. Lower inset: Close‐up of the inferior terminations of the CB‐V in the fusiform gyrus. (d) Illustration of the CB‐V fibers interconnecting the POS2, mid‐fusiform gyrus, and parahippocampal gyrus. Left hemisphere, infero‐medial view depicting the regional white matter anatomy following the micro‐dissection of the regional cortex and “U” fibers of the fusiform gyrus. Upper inset: Inset: The CB‐V is highlighted in red, SRF in yellow, and ILF in blue. Lower inset: Magnified view of the inferior terminations of the CB‐V reaching the middle part of the in the fusiform gyrus which corresponds to the fusiform face area. The trajectory of the CB‐V is outlined with black dotted arrows. CaF, calcarine fissure; dark red lines, CB‐V trajectory; dark red highlighted regions, CB‐V Cortical Projections; CaF, calcarine fissure; Gen, genu of the corpus callosum; FuG, fusiform gyrus; PPA, parahippocampal place area; PHG, parahippocampal gyrus; POS, parieto‐occipital sulcus; POS2, POS2 area of the precuneus; Spl, splenium of corpus callosum; TePo, temporal pole; Un, uncus.

After performing micro‐dissections of the cortex and underlying U‐fibers along the parieto‐occipital sulcus (POS), calcarine fissure, cuneus, precuneus, posterior cingulate cortex, retrosplenial complex, lingual gyrus, and posterior parahippocampal gyrus, we observed a distinct group of fibers connecting the posterior precuneus (POS2) and the parahippocampal place area (PPA). These fibers initially follow the trajectory of the POS and then bend around at the level of the retrosplenial cortex, curving caudo‐medially to reach the PPA (Figure 1b). The morphology and topography of this fiber tract correspond to the previously identified parietal parahippocampal cingulum (CB‐V) (Skandalakis et al., 2020).

The sledge runner fasciculus (SRF), can be identified traveling in an oblique rostro‐ventral direction from the anterior cuneus (posterior bank of POS) to the PPA, sharing termination points with the CB‐V. By performing cortical micro‐dissection of the collateral sulcus and selectively resecting the SRF and CB‐V fibers that terminate within the PPA, we observed fibers of the CB‐V passing deep to the level of the PPA These CB‐V fibers, curve laterally and fan out, ultimately terminating at the fusiform gyrus (Figure 1c).

**FIGURE 2:**
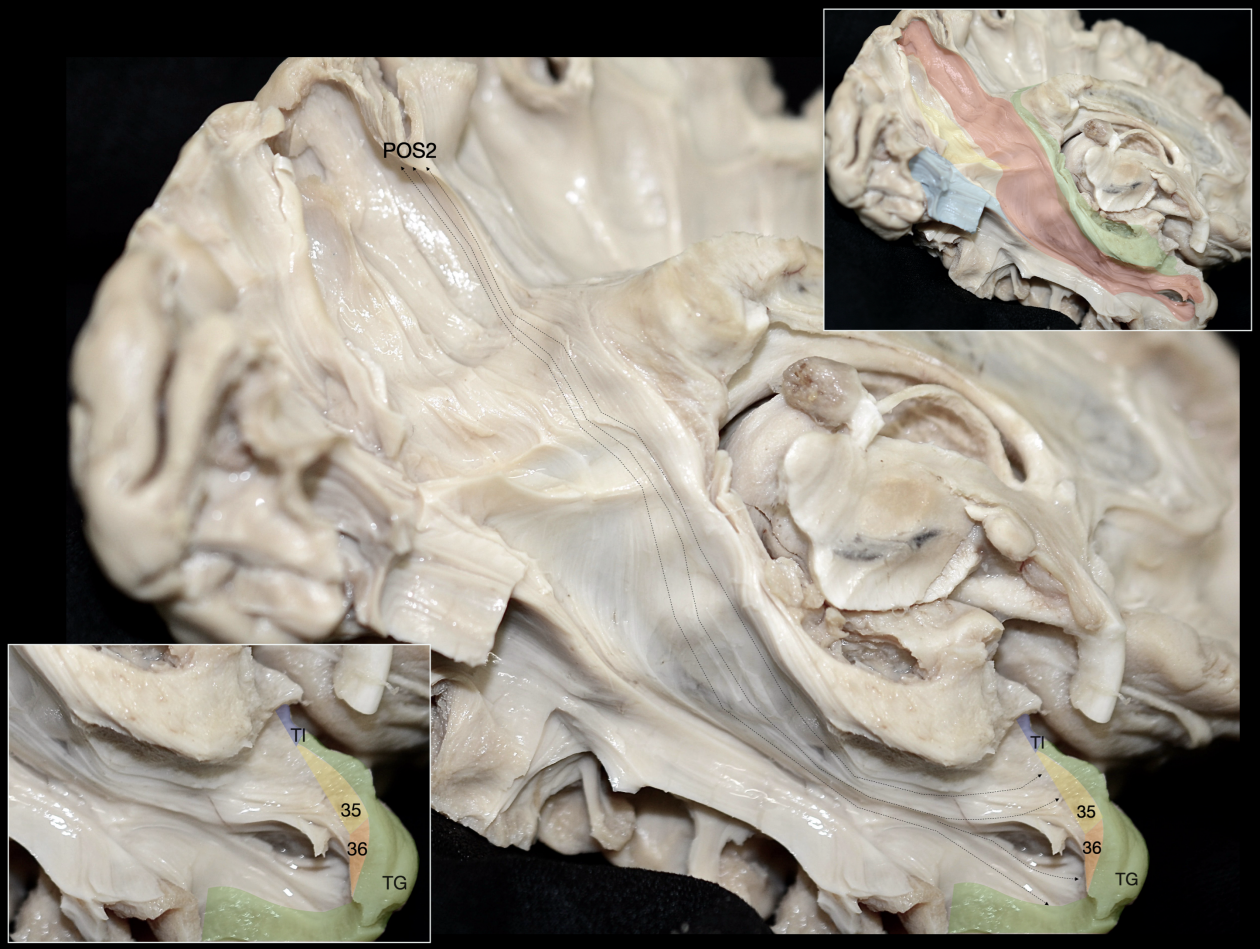
Illustration of the CB‐V fibers interconnecting the POS2 and TP. Illustration of the CB‐V fibers interconnecting the POS2 and areas 35 and TI of the TP. Left hemisphere, infero‐medial view demonstrating the fiber tract anatomy after resection of the inferior longitudinal fasciculus and CB‐V fibers terminating at the fusiform gyrus. Notice that fibers of the cingulum run posteriorly from rostral regions and then arch around the splenium of the corpus callosum at the mid‐sagittal plane whereas fibers of the CB‐V come from above and lateral and travel in an oblique direction, crossing the parasagittal plane. Upper left inset: Different angle highlighting the termination points to the POS2. Upper right inset: The CB‐V is highlighted in red, SRF in yellow, cingulum in green, and ILF (dissected) in blue. Lower inset: Magnified view of the inferior terminations of the CB‐V reaching the temporal pole.

**FIGURE 3:**
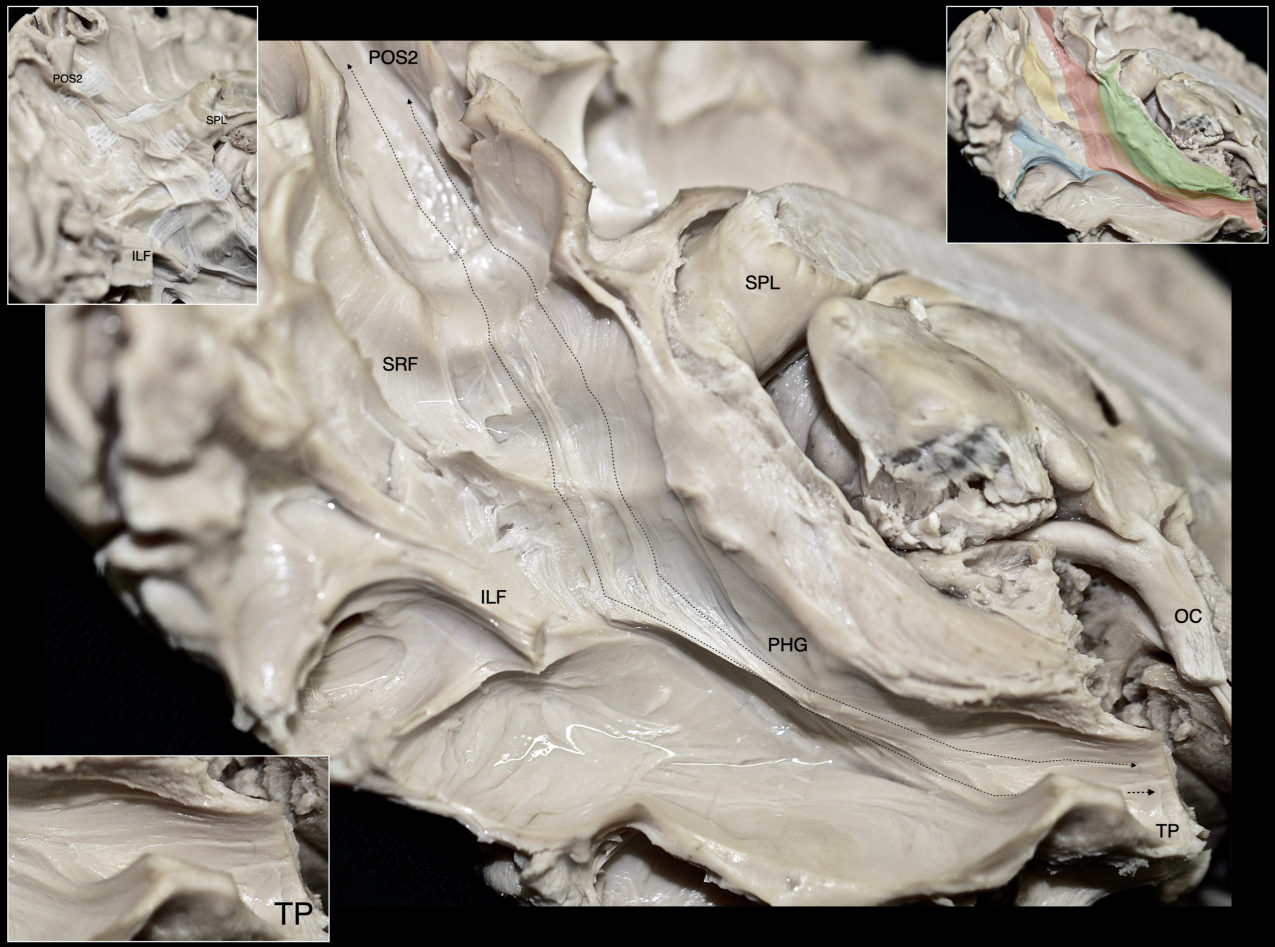
Illustration of the CB‐V fibers interconnecting the POS2 and parcellated regions of the Temporal Pole. Left hemisphere infero‐medial view demonstrating the regional fiber tract anatomy after dissection of the inferior longitudinal fasciculus, CB‐V fibers terminating at the fusiform gyrus, and cingulum fibers. Upper inset: Inset: The CB‐V is highlighted in red, SRF in yellow, cingulum in green and ILF (dissected) in blue. Lower inset: Magnified view of the inferior terminations of the CB‐V reaching the temporal pole. The trajectory of the CB‐V is outlined with black dotted arrows. Areas 35, 36, TI, and TG are highlighted in orange, yellow, blue, and green respectively. Gen, genu of corpus callosum; OC, optic chiasm; OcPo, occipital pole; PPA, parahippocampal place area; PHG, parahippocampal gyrus; SN, substantia nigra; Spl, splenium of corpus callosum.

**FIGURE 4:**
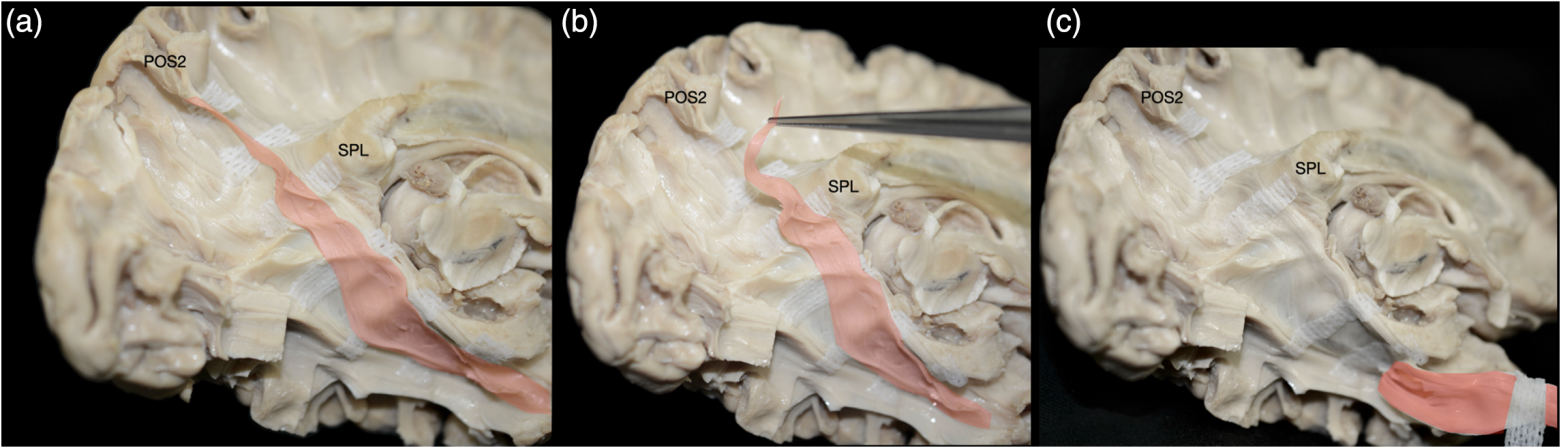
Continuity and structural integrity of CB‐V fibers. Fibers of the CB‐V are followed toward temporal pole. (a) Fibers of the CB‐V highlighted in red are dissected and separated from the hemisphere with tailored white gauze pads. (b, c) Fibers are progressively lifted with micro‐forceps to demonstrate their structural integrity which remains intact. Spl, splenium of corpus callosum; TePo, temporal pole.

**FIGURE 5:**
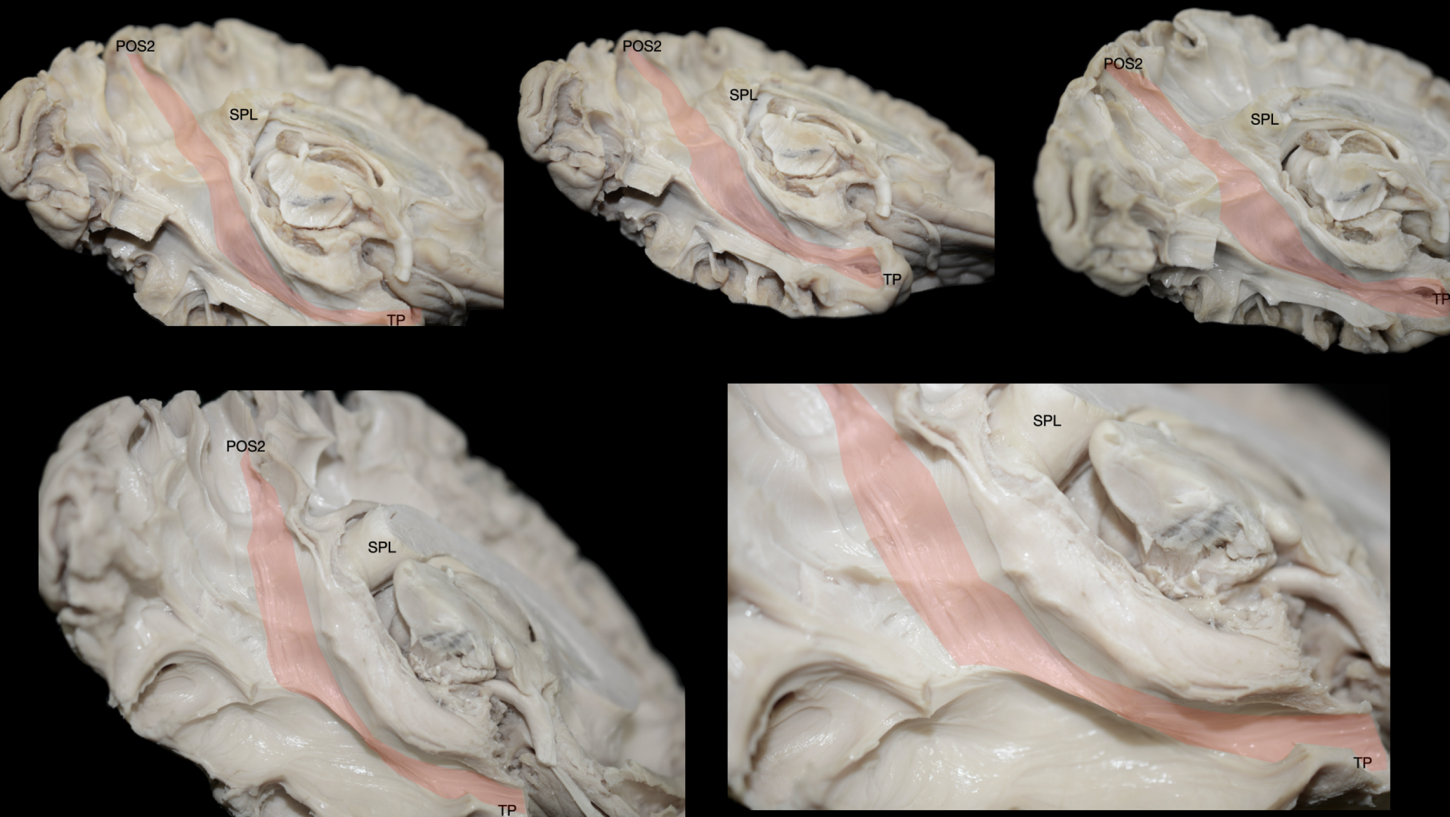
Additional views of the CB‐V. Fibers of the CB‐V interconnecting the POS2 with temporal pole highlighted in red captured through different angles. Spl, splenium of corpus callosum; TePo, temporal pole.

**FIGURE 6:**
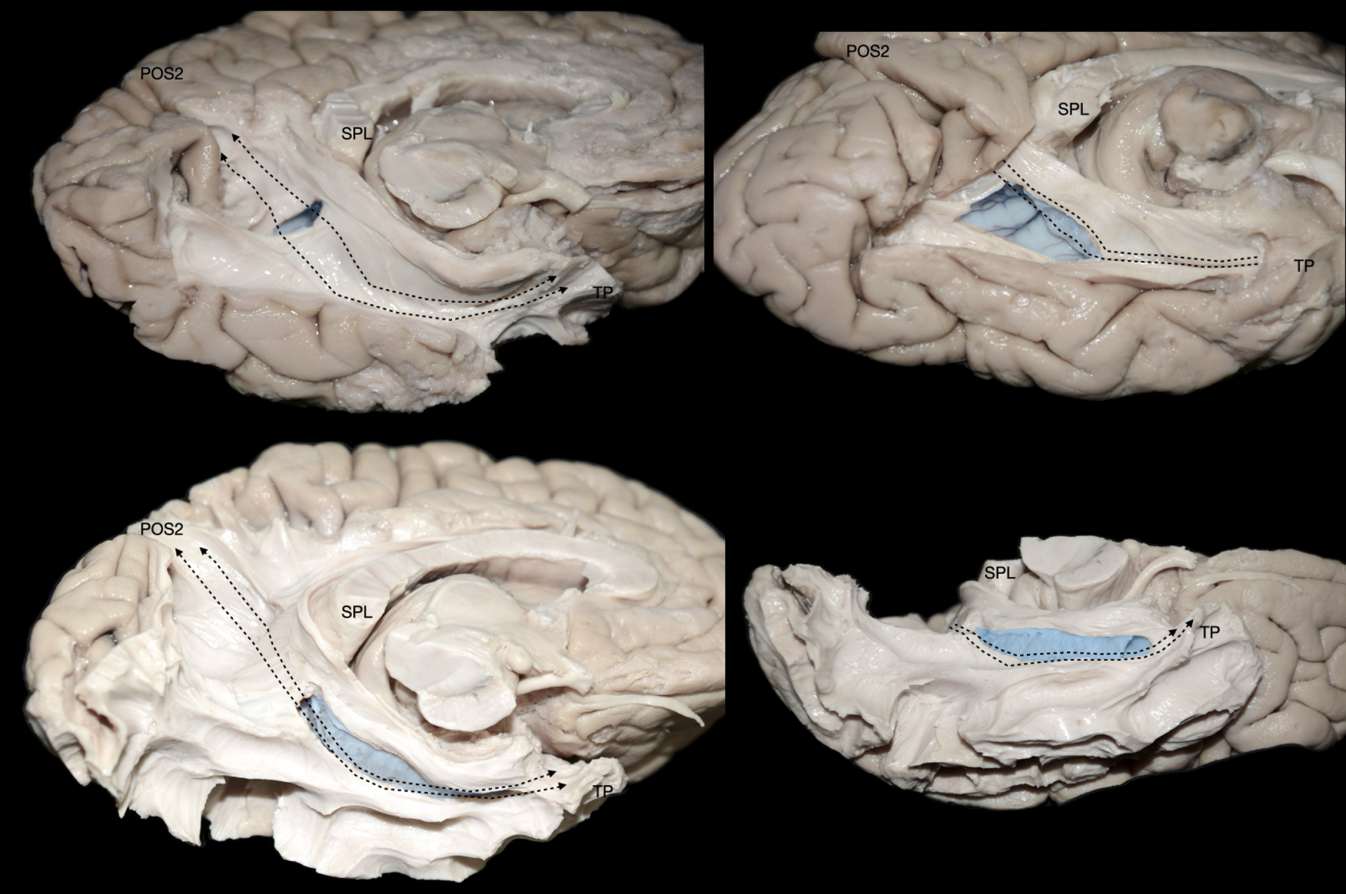
Spatial relationship of the CB‐V with the ventricular system. Dissections at different stages exposing the ventricular system at locations relevant to the CB‐V captured through different angles. Surgical windows expose the atrium and temporal horn highlighted in blue. The trajectory of the CB‐V which has been resected is illustrated with interrupted lines. Spl, splenium of corpus callosum; TePo, temporal pole.

Upon resection of the cortex and underlying U‐fibers of the fusiform gyrus, occipitotemporal sulcus, inferior occipital gyrus, and inferior temporal gyrus, we were able to visualize the ventral segment of the inferior longitudinal fasciculus (ILF) running between the occipital pole and TP. Additionally, fibers belonging to the CB‐V were observed terminating along the anterior two‐thirds of the ILF (Figure 1d). By meticulously resecting the CB‐V fibers in conjunction with the simultaneous resection of ILF fibers, we revealed a distinct group of fibers corresponding to a deeper layer of the CB‐V. These fibers exhibited a trajectory between the posterior precuneus (POS2) and the TP. Initially, they followed the path of the POS, widening and curving laterally at the level of the PPA. Notably, at the level of the ILF, these fibers projected anteriorly toward the temporal horn, narrowing as they reached the anterior part of the parahippocampal gyrus, and then fanning out again before reaching the TP (Figures 2, 3, 4, 5, 6).

### 3.2 Connectivity

Fibers exhibited continuity between the posterior precuneus (POS2) and the TP in all examined hemispheres. Significantly the fibers of the CB‐V reached the area POS2 of the precuneus as well as areas 35, 36, and TG of the TP in all of the examined hemispheres (Figure 2). Moreover, we observed fibers of the CB‐V reaching the area POS2 of the precuneus as well as areas 35, 36, TI, and TG of the TP in three out of nine hemispheres (Figure 3).

### 3.3 Adjacent pathways and structures

Fibers interconnecting POS2 and TP display a close relationship with fibers of the cingulum, SRF, forceps major, and ILF. More specifically we have recorded the following spatial characteristics of these neighboring tracts.

Fibers interconnecting POS2 and TP display a close relationship with fibers of the cingulum, SRF, forceps major, and ILF. More specifically, we have recorded the following spatial characteristics of these neighboring tracts.

Fibers of the cingulum originate from the frontal cortices and arch around the splenium of the corpus callosum with an almost parallel trajectory to the mid‐sagittal plane (Catani & De Schotten, 2008). Fibers of the CB‐V interconnecting the POS2 and TP originate at the posterior precuneus and run in an oblique dorsolateral to ventromedial direction, crossing the parasagittal plane (Figure 2). These fibers are observed in a more lateral and caudal location in relation to the main bundle of fibers of the cingulum which at this level (below the splenium of the CC) are defined as the inferior arm of the cingulum (Kadri et al., 2017). Fibers of both fascicles exhibit a close relationship below the level of the splenium, and as expected at the level of the parahippocampal gyrus and hippocampal formation they mingle.

The SRF is a fiber tract that also resides in the depth of the POS (Koutsarnakis et al., 2019). In contrast to CB‐V fibers which originate at the posterior precuneus (anterior bank of the POS), the SRF originates at the anterior cuneus (posterior bank of the POS) and travels in an oblique rostro‐ventral direction terminating at the PPA. CB‐V and SRF fibers are seen to cross the deep segment of the POS and share common termination points at the PPA. (Figure 1).

Fibers originating from the splenium of the corpus callosum are known as the forceps major or posterior forceps. They run parallel to the axial plane toward the occipital cortex, exhibiting a curve that resembles the convexity of the lateral surface of the cerebral hemisphere. Fibers interconnecting the POS2 and TP run in an oblique direction medial to the forceps major curving around its fiber; thus, displaying characteristic wave‐like configurations (Figures 2 and 3).

ILF fibers run in the axial plane at the depth of the fusiform gyrus. CB‐V fibers originate from the precuneus and terminate along the anteriormost fibers of the ILF or just medial to it, around the U fibers and cortex of the fusiform gyrus (Figure 1). Furthermore, longer CB‐V fibers, which continue toward the TP, travel superior to the ILF in a parallel direction.

### 3.4 Population‐based human tractography

Using a population‐based averaged template, we achieved successful reconstruction of the fiber tract that interconnects the posterior precuneus area (POS2) with the parahippocampal gyrus, areas 35, 36, TI, and TG (Figure 11). These reconstructed results align closely with the known structure, trajectory, and connectivity patterns of the CB‐V fibers that were observed during our meticulous micro‐dissections. The trajectory of the fibers followed the orientation of the anterior bank of the POS and the ILF. The fibers run medial to the atrium and fan out at the level of the anterior parahippocampal gyrus before reaching the TP. The length of the tact was 108.87 mm in the right hemisphere and 120.1 mm in the left hemisphere. The volume of the tract was measured 3227.38 mm^3^ in the left hemisphere and 3266.5 mm^3^ in the right hemisphere.

**FIGURE 7:**
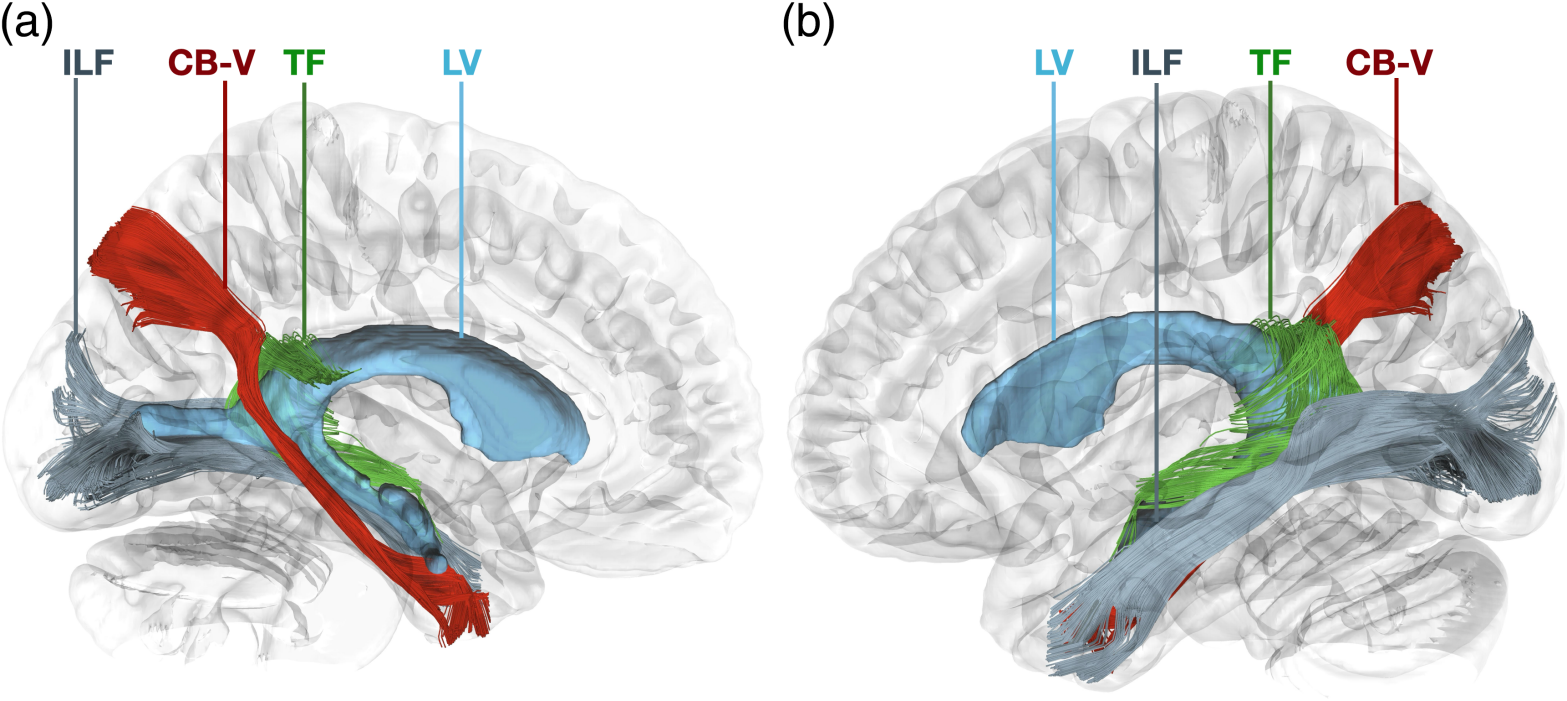
Anatomical detail of the lateral ventricle and relationship of the CB‐V with the tapetum, and the ILF. Left hemisphere reconstruction of the tapetum, ILF and CB‐V using the 1065 HCP averaged template on a (a) medial and (b) lateral view. The CB‐V runs medial to the atrium whereas the tapetum fibers, and ILF reside at the lateral side of the atrium and temporal horn of the lateral ventricle. CB‐V, cingulum bundle V; ILF, inferior longitudinal fasciculus; LV, lateral ventricle; TF, tapetum fibers.

**FIGURE 8:**
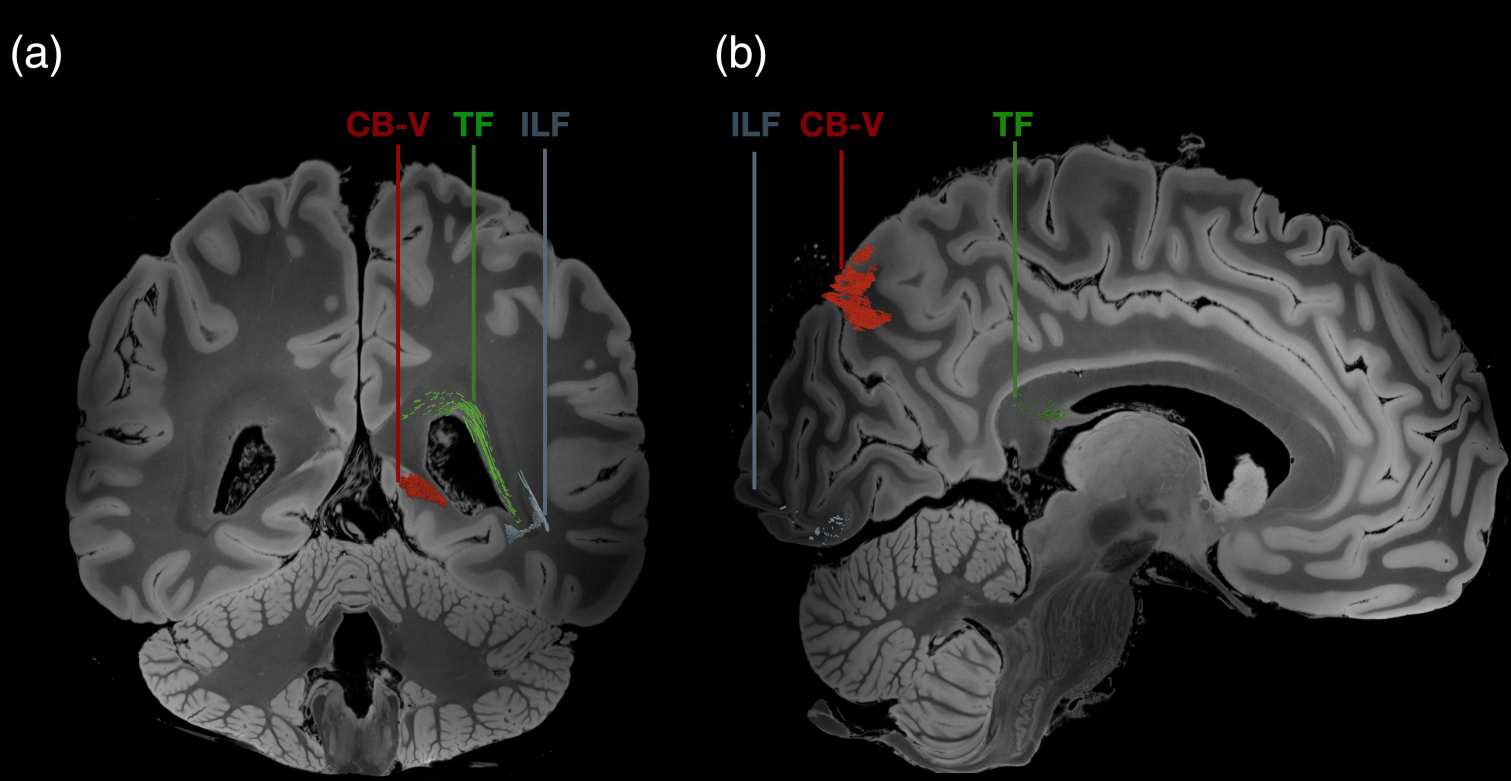
Relationship of the ventricular system with CB‐V, tapetum, and the ILF. Left hemisphere reconstruction of the tapetum, ILF and CB‐V using the 1065 HCP. (a) Coronal slice at the level of the atrium showing the CBV running at the medial side of the hemisphere whereas the tapetum fibers, and ILF reside at the lateral side of the atrium. (b) Sagittal slice at the level of the lateral ventricle showing fibers of the CB‐V running at the anterior bank of the parietooccipital sulcus corresponding to area POS2, tapetum fibers running within the splenium of the corpus callosum, and fibers of the lLF within the occipital pole.

**FIGURE 9:**
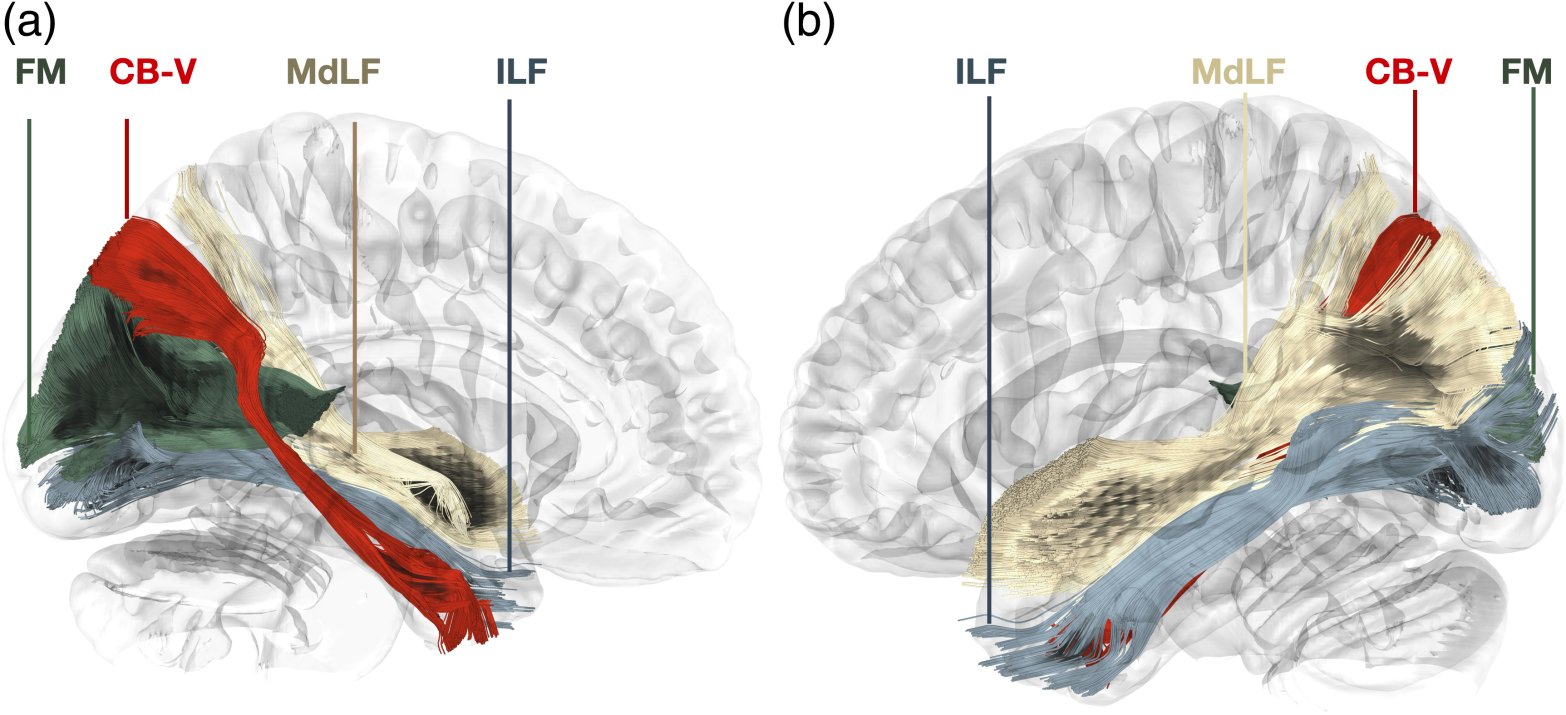
Relationship of the CB‐V with adjacent tracts. Left hemisphere reconstruction of the ILF, MdLF, Forceps major and CB‐V using the 1065 HCP averaged template on a (a) medial and (b) lateral view. Note that the forceps major has been cut on the level of the midline, and the segment of the tract that runs within the right hemisphere has been removed to allow for visibility of the medial view. The CB‐V runs medial to ILF, MdLF, and forceps major. CB‐V, cingulum bundle V; ILF, inferior longitudinal fasciculus; MdLF, middle longitudinal fasciculus; FM, forceps major.

**FIGURE 10:**
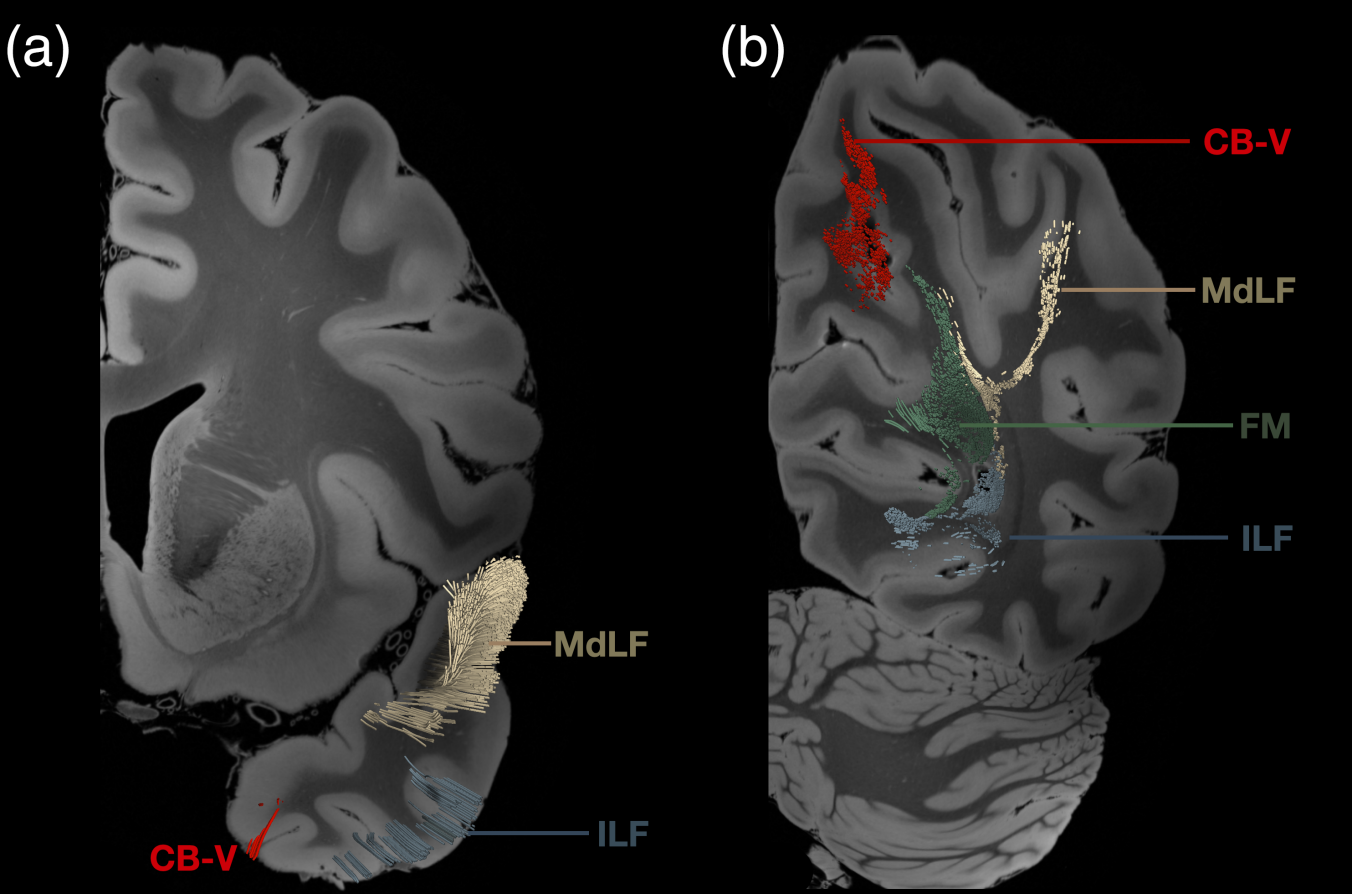
Termination points of the CB‐V and relationship with adjacent tracts. Left hemisphere reconstruction of the ILF, MdLF, Forceps major and CB‐V using the 1065 HCP averaged template. (a) Coronal slice at the level of the TP showing the of the termination points of the CB‐V, MdLF, and ILF. (b) Coronal slice at the level of the POS2 showing the of the termination points of the CB‐V within the POS2 and the relationship of the, MdLF, and ILF, and forceps major within the posterior segment of the sagittal stratum. CB‐V, cingulum bundle V; ILF, inferior longitudinal fasciculus; MdLF, middle longitudinal fasciculus; FM, forceps major.

The fidelity of our reconstructions was evident as we compared them to the data obtained through the micro‐dissection procedure. The extracted fiber tracts demonstrated remarkable consistency with the observed structure and trajectory of the CB‐V fibers. This agreement further supports the reliability and accuracy of our reconstruction methodology. To demonstrate the relationship of the CB‐V we adjacent tracts, we successfully reconstructed the ILF, Forceps major, MdLF, and tapetum fibers (Figures 7, 8, 9, 10).

**FIGURE 11:**
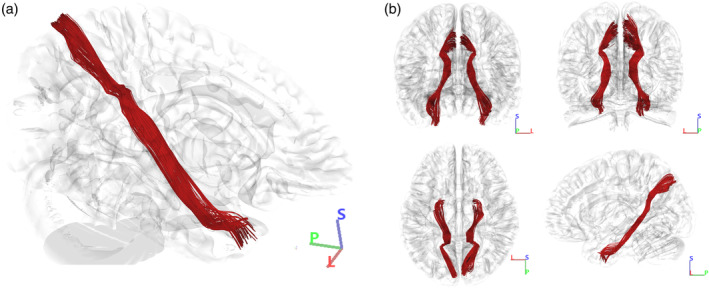
Population‐based human tractography reconstruction of the cingulum connections between POS2 and TP. (a) Inferomedial view of the reconstructed tracts demonstrating the similarities in structure, trajectory, and connectivity patterns of the CB‐V fibers recorded by our micro‐dissections. The fiber tract interconnects the posterior precuneus area (POS2) with the parahippocampal gyrus, areas 35, 36, TI, and TG. (b) Anterior, posterior, superior, and lateral views of our reconstructed fiber tracts.

**FIGURE 12:**
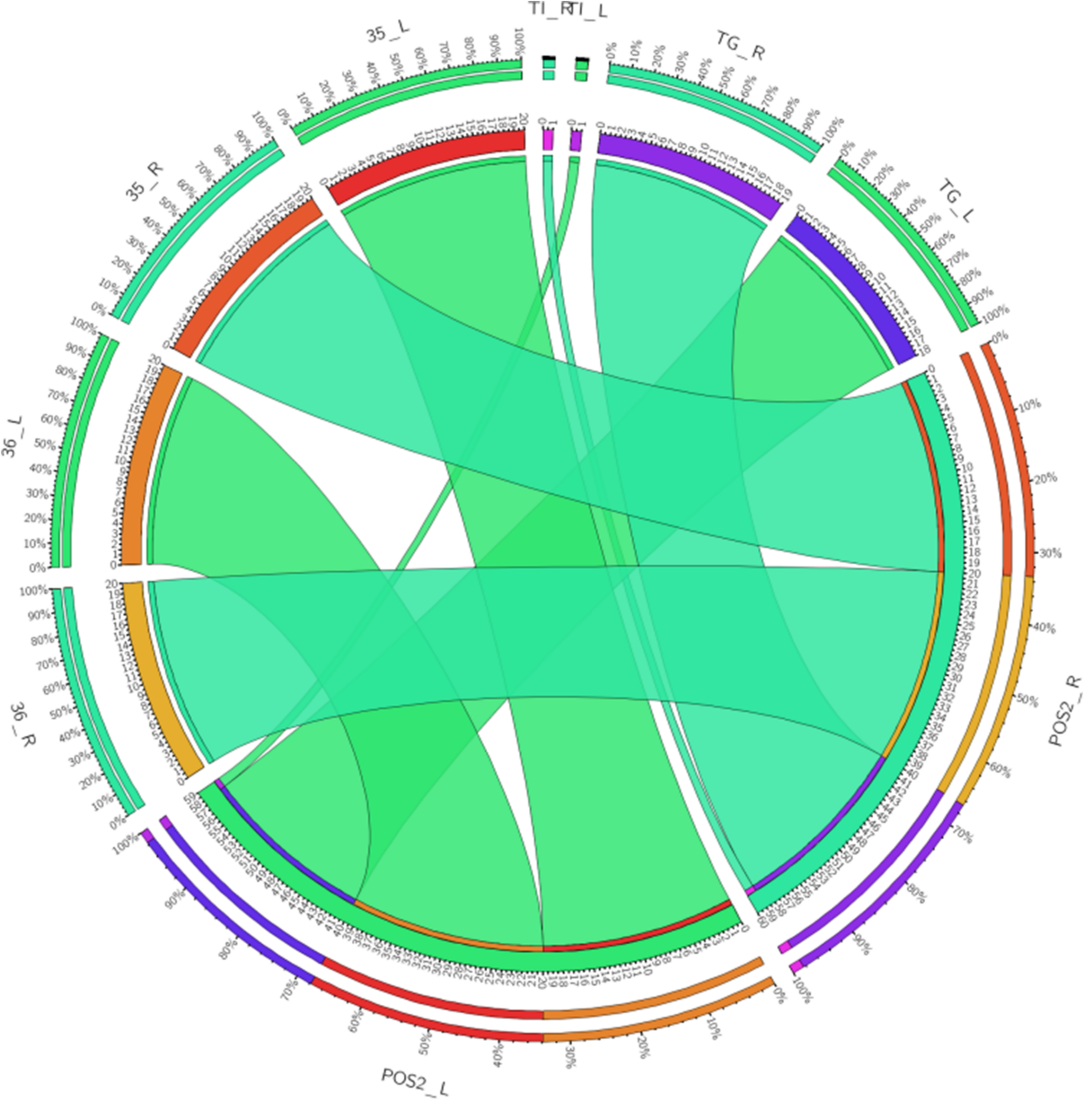
A connectogram representing bilateral connectivity of the CB‐V created using the CIRCOS data visualization tool. Areas of the temporal pole and precuneus studied are listed around the circumference of the connectogram. The suffix a suffix _L or _R represents Left and Right hemispheric connections.

**FIGURE 13:**
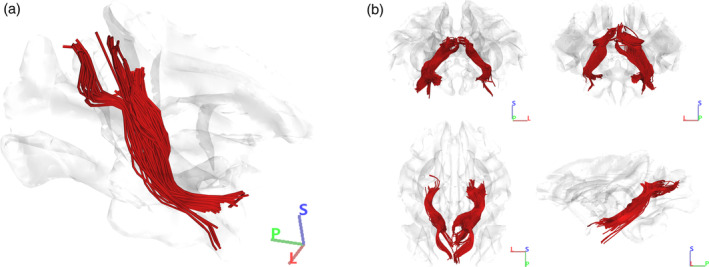
Population‐based rhesus macaque tractography of the cingulum connections between the medial parietal lobe and temporal pole. (a) Inferomedial view of the reconstructed tracts demonstrating the similarities in structure and trajectory of the CB‐V fibers recorded by our human micro‐dissections. (b) Anterior, posterior, superior, and lateral views of our reconstructed fiber tracts.

Overall, our findings provide robust evidence for the existence and connectivity of the fiber tract linking the POS2 with the parahippocampal gyrus areas, particularly area 35. Furthermore, our successful reconstruction of the fiber tracts connecting the POS2 with areas 35, 36, TI, and TG in further strengthens our understanding of the intricate connectivity within these brain regions.

### 3.5 Subject specific human tractography

We successfully reconstructed the fiber tract that interconnects the area POS2 with the TP in 40 hemispheres from 20 healthy subjects (Supporting Information). The subject‐specific connectivity between area POS2 and the different areas of the TP (36, 36, TI, and TG) is presented in Table 1 and Figure 12. All subjects exhibited bilateral connections between POS2 and areas 35, and 36. Bilateral connections between areas POS2 and TG were documented in 17 out of 20 subjects. Unilateral connections between areas POS and TG were documented in 3 out of 20 subjects (2 left and 1 right). Bilateral connections between areas POS2 and TI were documented in 1 out of 20 subjects. The quantitative data analyzed in 40 hemispheres from 20 individual healthy subjects from the human connectome project are summarized in Table 2. The mean length of the tact was 109.15 mmm (SD ± 12.64 mm) in the left hemisphere and 114.11 mm (SD ± 12.81 mm) in the right hemisphere. The mean volume of the tract was measured 3019.45 mm^3^ (SD ± 1133.15 mm^3^) in the left hemisphere and 3046.48 mm^3^ (SD ± 1150.99 mm^3^) in the right hemisphere. The mean volume lateralization index was 0 (SD ± 0.01). The mean fractional anisotropy was measured 0.42 (SD ± 0.03) in the left hemisphere and 0.42 (SD ± 0.04) in the right hemisphere.

**TABLE 1:** Qualitative connectogram. Termination points of the CB‐V in the areas POS2, 35, 36, TI, and TG.

Subject	Gender	Age	POS2		TG		TI		35		36	
			L	R	L	R	L	R	L	R	L	R
971160	M	26–30	+	+	+	+	−	−	+	+	+	+
970764	F	22–25	+	+	+	+	−	−	+	+	+	+
966975	M	22–25	+	+	+	+	−	−	+	+	+	+
969476	M	31–35	+	+	−	+	−	−	+	+	+	+
951457	F	31–35	+	+	+	+	−	−	+	+	+	+
959574	F	26–30	+	+	+	+	−	−	+	+	+	+
958976	M	26–30	+	+	+	+	−	−	+	+	+	+
952863	F	22–25	+	+	+	+	−	−	+	+	+	+
955465	M	26–30	+	+	+	+	−	−	+	+	+	+
965771	M	31–35	+	+	−	+	−	−	+	+	+	+
965367	M	31–35	+	+	+	+	−	−	+	+	+	+
962058	M	26–30	+	+	+	+	+	+	+	+	+	+
930449	F	22–25	+	+	+	+	−	−	+	+	+	+
932554	M	26–30	+	+	+	−	−	−	+	+	+	+
933253	F	26–30	+	+	+	+	−	−	+	+	+	+
937160	M	26–30	+	+	+	+	−	−	+	+	+	+
942658	M	26–30	+	+	+	+	−	−	+	+	+	+
943862	M	26–30	+	+	+	+	−	−	+	+	+	+
947668	F	26–30	+	+	+	+	−	−	+	+	+	+
927359	M	22–25	+	+	+	+	−	−	+	+	+	+

**TABLE 2:** Quantitative tractography data.

Subject	Gender	Age	Length		FA		Volume		
			L	R	L	R	L	R	LI
971160	M	26–30	118.46	120.78	0.45	0.47	1913.50	3808.88	0.33
970764	F	22–25	77.99	115.53	0.41	0.38	2426.75	2329.38	−0.02
966975	M	22–25	116.58	132.23	0.46	0.43	2181.00	4375.50	0.33
969476	M	31–35	100.69	135.25	0.41	0.40	712.88	2800.25	0.59
951457	F	31–35	98.45	101.78	0.47	0.52	4457.25	3129.50	−0.18
959574	F	26–30	112.42	117.54	0.45	0.45	3993.75	3072.00	−0.13
958976	M	26–30	110.49	117.07	0.40	0.38	1911.62	1371.50	−0.16
952863	F	22–25	100.84	105.31	0.40	0.42	1973.00	2158.50	0.04
955465	M	26–30	114.59	109.20	0.38	0.40	4040.75	3871.38	−0.02
965771	M	31–35	113.99	115.83	0.39	0.37	3245.38	4923.00	0.21
965367	M	31–35	125.92	136.03	0.45	0.39	4573.75	2243.88	−0.34
962058	M	26–30	117.52	119.39	0.44	0.43	2859.75	4348.62	0.21
930449	F	22–25	90.50	104.79	0.43	0.43	2265.75	3174.25	0.17
932554	M	26–30	99.54	91.90	0.44	0.40	2326.38	751.38	−0.51
933253	F	26–30	114.40	117.65	0.42	0.41	3558.75	5022.12	0.17
937160	M	26–30	103.06	115.23	0.44	0.42	5380.62	3739.00	−0.18
942658	M	26–30	115.98	116.07	0.43	0.45	3594.12	2447.75	−0.19
943862	M	26–30	135.08	122.93	0.41	0.39	3471.50	2192.00	−0.23
947668	F	26–30	104.59	91.30	0.37	0.38	2344.12	1905.38	−0.10
927359	M	22–25	111.96	96.44	0.38	0.41	3158.38	3265.25	0.02
Mean			109.15	114.11	0.42	0.42	3019.45	3046.48	0.00
SD ±			12.64	12.81	0.03	0.04	1133.15	1150.99	0.01

### 3.6 Rhesus macaque fiber tractography

Using a rhesus averaged template, we conducted a comprehensive analysis of fiber tracts connecting the medial parietal and temporal lobe through the parahippocampal cingulum, resulting in successful reconstruction (Figure 13). The fiber tracts exhibit a continuous and distinct course along the medial surface, closely resembling the structure and trajectory observed in human cingulum fibers.

## 4 DISCUSSION

In the present study, we performed focused parcellation‐based micro‐dissections to investigate the fiber tracts connecting precuneus and TP. Our study revealed a distinct group of fibers interconnecting the POS2 with areas 35, 36, and TG. These fibers belong to the CB‐V system and exhibit a very close relationship with the cingulum, SRF, forceps major, and ILF. Utilizing tractography, we successfully reconstructed the fiber tract connecting precuneus with TP, which closely matched our micro‐dissection findings. We found connectivity between POS2 and TI in the minority of the subjects. This finding was recorded in only three out of nine hemispheres and in only 1 out 20 single subjects analyzed. Previous studies have investigated the connectivity between the parietal and temporal lobe (Vos de Wael et al., 2021). The structural connectivity between precuneus and basal‐medial temporal lobe has been reported by means of the cingulum (Jung et al., 2017). The connectivity of the precuneus with areas TA, and TG of the TP has been demonstrated by means of the MdLF (Kalyvas et al., 2020; Sasaki et al., 2023). To our knowledge, this is the first comprehensive study that provides detailed insights into the *structural* connectivity between POS2, area 35, 35, TI, and TG, facilitated by CB‐V fibers. Overall, our study enhances our understanding of the intricate connectivity patterns within these brain regions and provides valuable insights into the organization of fiber tracts within the human brain.

### 4.1 Human connectivity

Previous studies utilizing resting‐state fMRI have demonstrated strong functional connectivity among the posterior precuneus, parahippocampal gyrus, and areas TG and TI of the TP (Fan et al., 2014; Pascual et al., 2015). The term “parahippocampal cingulum” has been used by Jones et al. to describe a bundle of fibers connecting the posterior precuneus, posterior cingulate cortex, and medial temporal lobe (Jones, Christiansen, et al., 2013). Wu et al. introduced the term “Cingulum Bundle‐V" to describe the segment of the cingulum connecting the precuneus with the ventral and medial temporal lobe (Wu et al., 2016). Tanglay et al. referred to the CB‐V as the “parahippocampal cingulum” in their investigation of precuneus connectivity, describing it as a group of fibers traveling from the precuneus and terminating in the parahippocampal gyrus (Tanglay et al., 2022). Additionally, Jitsuishi and Yamaguchi reported the existence of a fiber bundle connecting the posterior precuneus and medial temporal lobe using a combination of fiber micro‐dissections and imaging techniques (Jitsuishi & Yamaguchi, 2021). Our previous study focused on the connectivity of the precuneus and temporal lobe (Skandalakis et al., 2020), but did not specifically investigate the connectivity of the TP. Therefore, our current study expands upon the existing literature by elucidating the connectivity patterns of the CB‐V in relation to the TP, providing valuable insights into the organization of this fiber tract and its role in connecting key brain regions involved in memory and spatial processing. The TP is a complex region implicating several different fiber tracts such as the uncinate fasciculus (UF), ILF, middle longitudinal fasciculus fiber, anterior limb of the anterior commissure, inferior thalamic radiations, and temporal limb of the ansa peduncularis (Charalampopoulou et al., 2022; Kalyvas et al., 2020; Liakos et al., 2021; Sahin et al., 2023). Studies investigating the connectivity of the aforementioned fiber tracts within distinct subregions of the temporal lobe are limited. Imaging studies using DTI with probabilistic tractography report connectivity of the area TG through the UF and ILF (Fan et al., 2014). Future studies should elucidate the connectivity of each subregion of the temporal lobe and the potential overlap of termination points.

### 4.2 Rhesus macaque connectivity

Our rhesus macaque averaged template results demonstrated the presence of a continuous and distinct tract along the medial surface interconnecting the precuneus and TP. Nevertheless, connectivity tract‐tracing studies of the precuneus and TP in non‐human primates have not demonstrated a connection between the two (Baizer et al., 1991; Bakola et al., 2010; Bakola et al., 2013; Bakola et al., 2017; Gamberini et al., 2020; Passarelli et al., 2018; Jones & Powel 1969; Markowitsch et al., 1985; Pandya & Kuypers, 1969; Pandya & Seltzer 1982; Passarelli et al., 2011). Tracer studies report that the continuity of these fiber tracts is disrupted at the level of the retrosplenial cortex, suggesting the presence of anatomical discontinuities or alternative pathways in this region (Table 3). As such, our animal data should be interpreted with caution. Focused tracer or klingler's micro‐dissection animal studies should investigate anatomy of the parahippocampal cingulum to clarify whether the association tract connecting the area POS2 with regions of the TP is a fiber tract unique in humans. Cross species differences may be due to the more complex cytoarchitecture of the human precuneus and TP which exhibit connections unique to the human brain as well as the differences in connectivity and functional organization between species (Cavanna & Trimble, 2006; Insausti, 2013). The human precuneus is more vastly connected with the fusiform gyrus and regions of the temporal lobe located more ventrally and anteriorly in comparison to the macaque; this circuit whose functional role has likely reformed over the course of evolution subserves memory, planning, and spatial navigation (Hutchison et al., 2015).

**TABLE 3:** Relevant projections of the precuneus and temporal pole in non‐human primates.

Study	Species	Method	Initial injection	Relevant projection	Reported projections
Passarelli 11	Macaca fascicularis (*n* = 5)	Fluorescent tracers retrograde	V6a1	MT, MST	Mesial Parietalcx, Frontal cx, intraparietal cx/inferior parietal lobule, Visual cx
Bakola '13	Macaca fascicularis (*n* = 5)	Fluorescent tracers retrograde	PE (posterior parietal cortex)	X	Mesial Parietal cx, Frontal cx, Somatosensory cx, Lateral Parietal cx
Bakola '10	Macaca fascicularis (*n* = 7 hemispleres, 6 subjects)	Fluorescent tracers retrograde	PEc	X	Mesial Parietal cx, Frontal cx, Somatosensory cx, Lateral Parietal cx
Passarelli '18	Macaca fascicularis (*n* = 5)	Fluorescent tracers retrograde	PGm (7 m)	MT− MST++ TPO/TA+/++ Tpt+ Parahip +−	PFV, PM/M, limbic, inf parietal, intraparietal, sup parietal, mesial, visual
Bakola '17	Macaca fascicularis (*n* = 5)	Fluorescent tracers retrograde	Area 31	MT− MST+ TPO/TA++ Tpt+++ Parahip +−	PFV, PM/M, limbic, inf parietal, intraparietal, sup parietal, mesial, visual
Pandya & Seltzer 82	Macaca mulatta *n* = 1	Tracers 3H proline/leucine	PGm, PE PEc	Tpt, sts, PGop	
Leichnetz '01	Cebus paella *n* = 1, Macaca fascicularis *n* = 2	HRP	7m	MST PGop	7a, 7ip (LIP, MIP, VIP, FEF, SEF)
Borra '10	Macaca mulatta *n* = 3	Fluorescent tracers retrograde	TE	X	TEO, V4, Tep, STS, IPS, PFC, perirhinal temporal pole
Pandya & Kuypers '69	Macaca mulatta *n* = 24 (one temporal pole)	Lesions/degeneration	TG	X	TE, STG (efferents)
Baizer '91	Macaca mulatta *n* = 5 macaca fascicularis *n* = 1	WGA‐HRP, florescent tracers, 3H aminoacids	aVMT	X	Inferior temporal cx (TE TEO) (efferents)
Suzuki & Amaral '94	Macaca fascicularis *n* = 20 (4 inj at dorsal 36rm, 6 inj atventral 36r)	WGA‐HRP, retrograde fast blue, diamidino yellow	36r	X	TE TEO (afferents)
Jones & Powell 1970	Macaca mulatta *n* = 23 (1 tp)	Lesions/degeneration	Temporal pole	X	TE STG (afferents) also frontal lobe, TH
Markowitsch '85	Macaca mulatta *n* = 6, marmosets *n* = 6, squirrel monkeys *n* = 2	WGA‐HRP	Temporal pole	X	ITG (TE) STG, also prefrontal, insular and minor density cingulate amygdala basal forebrain (afferents)

The TP and the precuneus are regions exhibiting differences in their structural anatomy and function between species (Glasser et al., 2014; Schleicher et al., 1999; Van Essen & Dierker, 2007). Differences in the connectivity and functional organization of the precuneus across species have been documented and suggest that the functional role has likely changed over the course of evolution (Hutchison et al., 2015). Gyrification of the TP seems to be higher in humans by the presence of two polar sulci which are not present in non‐human primates (Blaizot et al., 2010). Human TP connectivity is more extensive and localized more anteriorly when compared with chimpanzees and macaques (Bryant et al., 2019). Moreover, the human TP exhibits increased connectivity with parietal regions compared to non‐human primates (Pascual et al., 2015). It is suggested that the augmented connectivity of the human temporal lobe with the parietal lobe may be associated with the high‐order functions that are unique in humans (Catani, 2022). TP subregions of non‐human primates have distinct anatomical connections with regions of the temporal lobe, prefrontal cortex, and insula (Table 3) (Borra et al., 2010; Nakamura & Kubota, 1996). Conversely, posterior parietal regions of non‐human primates are mostly connected to primary somatosensory and motor cortices, cingulate cortex and areas of the occipital lobe (Gamberini et al., 2020).

### 4.3 Functional implications

The precuneus and medial temporal lobe are regions that are implicated in higher‐order brain function. (Dadario & Sughrue, 2023; Mesulam, 2023). Data from functional imaging studies show that area POS2 participates in visual, and auditory tasks activated in language domains but also in cognitive and emotional tasks involving social cognition (Luo et al., 2020). The TP is gaining support as one of the neural systems subserving spatial perception and autobiographical memory (Kalyvas, 2021; Setton et al., 2022; Teghil et al., 2021), brain functions traditionally related to the precuneus. Moreover, the TP has been implicated in memory, emotional processing, and face perception (Liu, Bernhardt, et al., 2016; Liu, Wang, et al., 2016; Olson et al., 2007). The TP can be considered a cortical convergence hub where social and emotional inputs are integrated (Mesulam, 2023; Pascual et al., 2015; Pehrs et al., 2017). The functional connectivity of the precuneus and parahippocampal gyrus is implicated in verbal creativity and episodic memory (Sun et al., 2018; Wade‐Bohleber et al., 2018). Both the precuneus and TP are large association areas subserving a vast array of high‐order functions. Studies on the parcellation of the precuneus and TP show that their subregions exhibit a distinct cytoarchitecture pattern and are implicated in different functions (Ding et al., 2009; Luo et al., 2020). The CB‐V fibers interconnecting POS2 and TP shared termination cortical points with ILF and the main bundle of the Cingulum (inferior arm) suggesting that this tract is likely involved in more complex neural processing involving the ILF and cingulum circuits, which subserve the connectivity of the occipital and frontal lobe, respectively (Young et al., 2021). The existence of the axonal connectivity between the POS2 and medial TP provides original anatomical evidence about the direct connectivity of cortical areas that are heavily implicated in the neural circuit of core cognitive functions such as face and word recognition, facial expression perception, spatial navigation, visuospatial perception memory, and imagery. This constant anatomo‐functional “dialogue” between these regions through discrete white matter pathways, provides useful insights into the adjustment and integration of the neural inputs and correlates of complex social cognition. Future studies may bear insight in this interesting topic and therefore clarify the functional role of the connection between POS2 and TP in healthy subjects.

### 4.4 Clinical implications

The functional connectivity of parietal and temporal regions is disrupted in Alzheimer's disease, Parkinson's disease, epilepsy, schizophrenia, schizotypal personality disorder, and major depression disorder (Cheng et al., 2018; De Schipper et al., 2018; Koch et al., 2022; Liu et al., 2018; Liu, Bernhardt, et al., 2016; Young et al., 2018; Zhu et al., 2017). Stimulation of the precuneus by repetitive transcranial magnetic stimulation (rTMS) in healthy individuals modulates the connectivity between the precuneus and TP (Mancini et al., 2017). Results of a recently published randomized, sham‐controlled trial showed that rTMS of the precuneus may slow down cognitive and functional decline in patients with Alzheimer's disease by strengthening the connectivity between the precuneus and temporal lobe (Koch et al., 2022). In the same vein, a randomized multiple baseline study showed that rTMS of the precuneus in patients with Alzheimer's disease can improve cognitive function (Traikapi et al., 2022). To the best of our knowledge, intraoperative or lesioning data on the CB‐V remain scarce. Studies reporting data on intraoperative electrical stimulation mapping collectively suggest that the UF and ILF play a pivotal role in language processing, particularly in tasks associated with semantic processing and naming (Koay et al., 2022; Mandonnet et al., 2007). Intraoperative lesioning of the ILF has been reported to result in prosopagnosia (Young et al., 2021). Studies reporting data after intraoperative lesioning of the UF indicate deficits in the recognition and naming of objects and famous individuals (Papagno et al., 2011).

Corticothalamic networks have been suggested to play a key role in the pathophysiology of epilepsy (Ji et al., 2015). Nevertheless, extra‐thalamic networks have been also identified during seizures (Handforth et al., 2005). Precuneal epileptic manifestations can entail alterations in consciousness, sensory phenomena like illusions or hallucinations, motor symptoms such as focal or generalized tonic–clonic seizures, cognitive symptoms including memory disturbances or déjà vu experiences, and autonomic symptoms like changes in heart rate or sweating (Al‐Ramadhani et al., 2021; Harroud et al., 2017; Mailo & Tang‐Wai, 2015). The connectivity of the precuneus with the fusiform gyrus (Skandalakis et al., 2020) and motor/premotor areas (Komaitis et al., 2019) could possibly explain motor and visual semiology during seizures as demonstrated by electrophysiological data (Umeoka et al., 2007). Given the very high incidence of mesial temporal lobe epilepsy (Engel, 2001) precuneal seizures could originate from mesial temporal regions. Precuneal seizures can present with mesial temporal lobe symptoms (Elisevich et al., 2022). Accordingly, the fiber tract connecting the POS2 and TP may represent a structural substrate for occipital, motor extra‐thalamic, and mesial temporal lobe seizures. Given the strong recommendation of precuneus sampling in patients with temporal lobe epilepsy symptoms (Jaafar et al., 2022), this pathway should be considered for sampling in addition to pulvinar pathways during the investigation of epileptogenic networks in patients with mesial temporal lobe, occipital and/or motor symptoms. Future studies focused on patients suffering from neurological and psychiatric conditions may uncover their underlying pathophysiology and determine the role of connectivity between POS2 and TP in these disorders.

### 4.5 Limitations

One of the main limitations of our study is inherent to Klingler's technique itself. The fiber micro‐dissection technique is highly operator‐dependent and as such, prone to human error. Considering this limitation, dissections in our study were carried out by three different authors independently. Furthermore, the fiber micro‐dissection technique provides lower spatial resolutions when compared to histology and optical coherence tomography (Wang et al., 2011) and lower structural relationship accuracy during the investigation of crossing/kissing fiber tracts (Kalyvas et al., 2020). As such, this technique is not an appropriate technique to compare volume of tracts or suggest a lateralization pattern. Moreover, the fiber micro‐dissection technique cannot be used to study the origin of a tract. The fiber micro‐dissection technique in turn displays several advantages. This technique involves the fixation of cadaveric specimens in a formalin solution followed by a freeze—thaw process, during which the formation of ice crystals separates the white matter fibers; thus, facilitating their dissection and recording. It preserves axonal integrity and ultrastructure as shown by transmission electron microscopy‐derived evidence, thus allowing the investigation of the termination and connectivity pattern of the subcortical fiber pathways through the cortex‐sparing technique as applied in this study (Martino et al., 2011; Zemmoura et al., 2016). As such, it stands as one of the “gold standard” methods used to validate data deriving from DWI‐based tractography, as the latter is reported to suffer from inherent limitations particularly when kissing, bending, and crossing fiber populations are explored (Fernandez‐Miranda et al., 2012; Jones, Knösche, & Turner, 2013; Le Bihan et al., 2006; Oouchi et al., 2007; Thomas et al., 2014; Tournier et al., 2012; Vos et al., 2011; Yendiki et al., 2022). The CB‐V resides in the depth of the POS and medial temporal lobe regions, which are extensively occupied by many fibers belonging to other known fiber tracts such as the SRF, ILF, and forceps major. The UF is part of the external capsule and the anterior commissure part of the sagittal stratum. Both fiber tracts reside within the lateral aspect of the hemisphere and cannot be dissected through the medial to lateral technique. Given that the UF and anterior commissure are not dissected in this study, we cannot exclude that there is an overlap with CB‐V fibers within the temporal lobe. It is important to note that another limitation of this study is the absence of reported age information regarding the donors of the hemispheres used for fiber micro‐dissections.

### 4.6 Conclusion

This study demonstrates the direct axonal connectivity of area POS2 of the posterior precuneus with areas 35, 35, and TG of the TP through a distinct long association fiber tract. Fibers of this tract belong to the CB‐V pathway and exhibit a very close relationship with the cingulum, SRF, forceps major, and ILF. Utilizing population‐based human and rhesus macaque tractography, we successfully reconstructed the fiber tract connecting precuneus with TP, which closely matched our micro‐dissection findings. These findings suggest axonal connectivity unique within the human brain and support the differences in neural networks between species. Our results refine the current knowledge of structural brain connectivity and organization in terms of cortical and subcortical axonal interactions. Moreover, useful insights are provided, allowing the study of high‐level functioning circuits residing in the posteromedial cortex in greater detail; thus, setting the ground for a more thorough understanding of these networks in normal brain function and pathological conditions of the brain.

## AUTHOR CONTRIBUTIONS

**GPS, FCY, SK, EN, ED, SFK, DD, CGH, PNK, GZ, GS, CK, MK, AK:** Conception and design. **GPS, SK, EN, ED, DD, CGH, PNK, GZ, GS, CK, AK, FCY:** Acquisition of data. **GPS, SK, SFK, EN, ED, CGH, PNK, GZ, GS, CK, LTE, AK:** Analysis and interpretation of data. **GPS, SK, EN, ED, DD, CGH, PNK, GZ, GS, CK, LTE, AK:** Drafting the article. **GPS, SK, EN, ED, DD, CGH, PNK, GZ, GS, CK, FCY, MK, AK, LTE, WJL:** Critically revising the article. All authors: Reviewed submitted version of manuscript. **GPS, SK, EN, ED, DD, CGH, PNK, GZ, GS, CK, MK, FCY, LTE, AK:** Study supervision.

## CONFLICT OF INTEREST STATEMENT

Constantinos Hadjipanayis is paid consultant for Synaptive Medical, Stryker Corporation, and Hemerion Therapeutics. He has conflicts related to the manuscript.

## Supporting information


**Data S1:** Supporting Information.

## Data Availability

Imaging data used in this study are publicly available through the human connectome project (https://www.humanconnectome.org/) and DSI studio (https://dsi-studio.labsolver.org/). Cadaver data are not publicly available due to privacy reasons.
