# Supplementary figures and images for: Unveiling the axonal connectivity between the precuneus and temporal pole: Structural evidence from the cingulum pathways
Source: Hum Brain Mapp. 2024 Jun 25;45(9):e26771. doi: 10.1002/hbm.26771 (PMC11199201; doi:10.1002/hbm.26771)

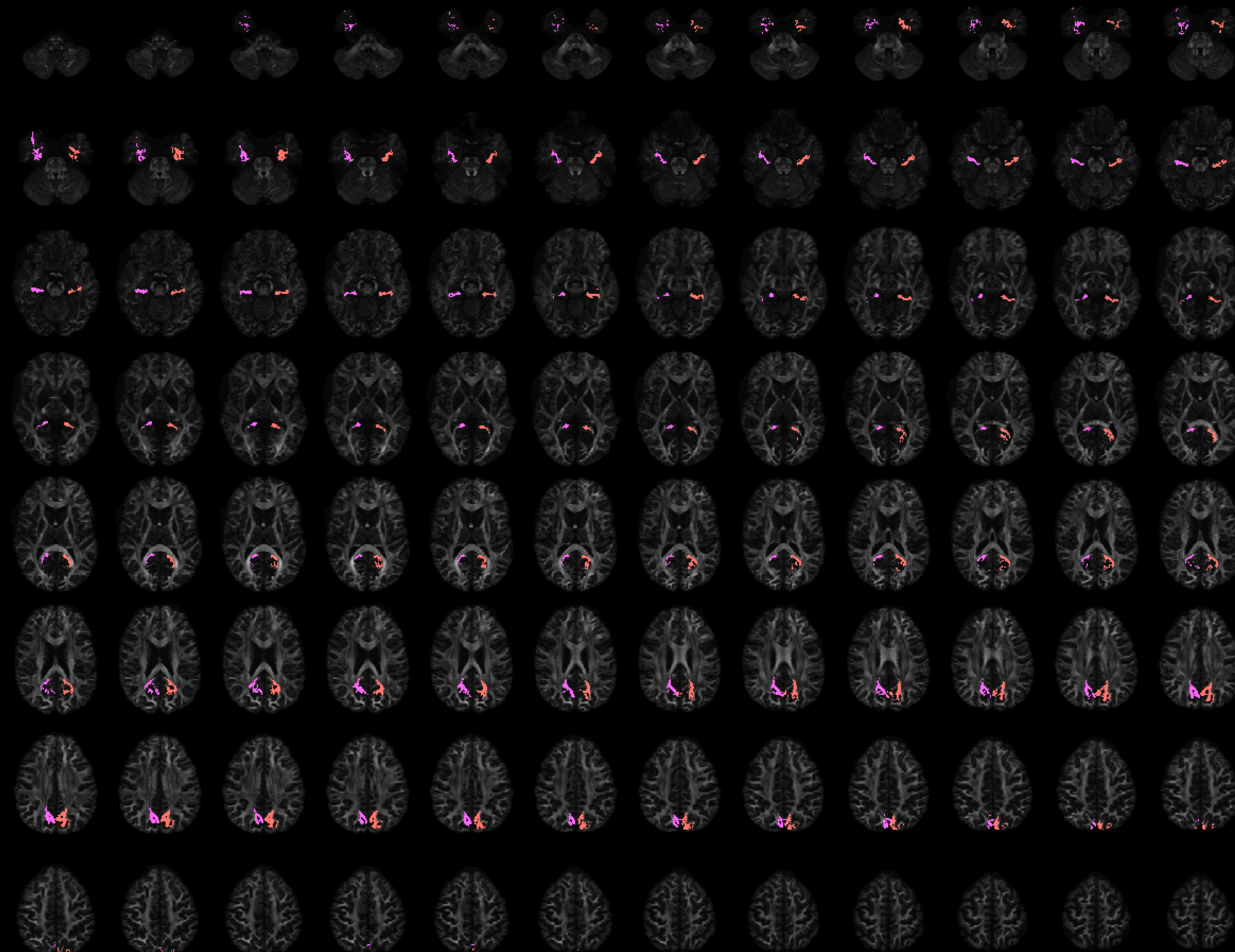

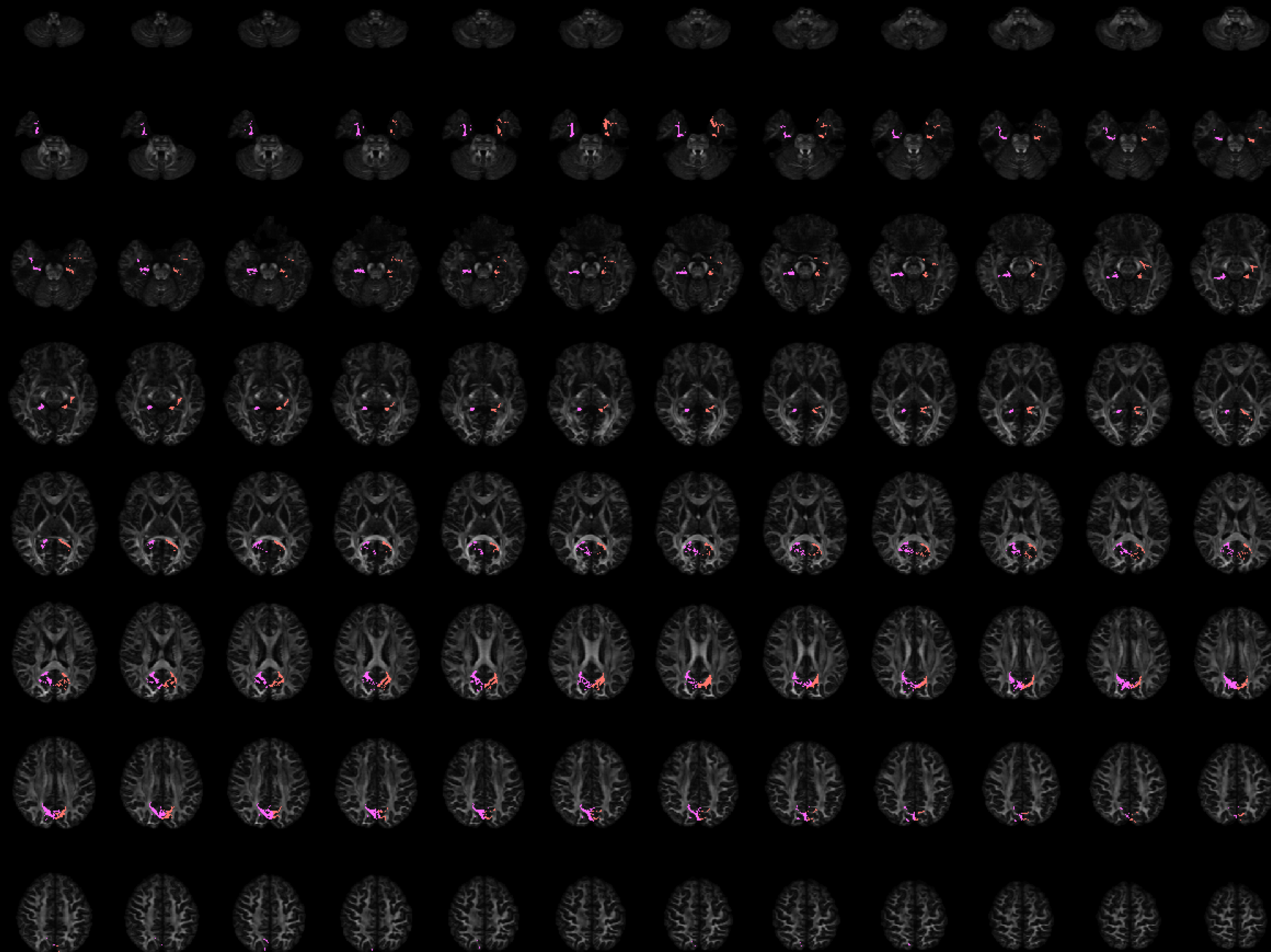

R 971160

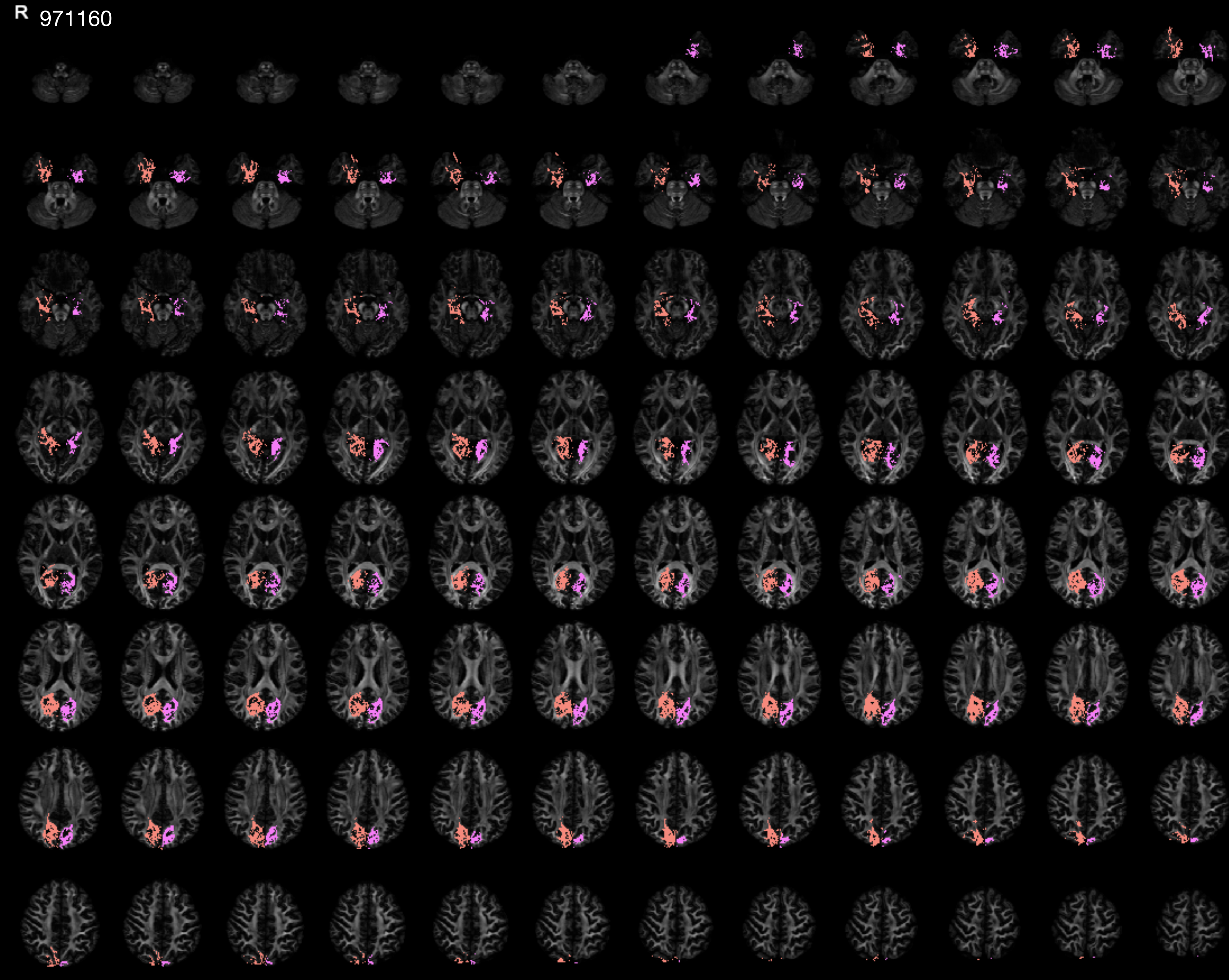

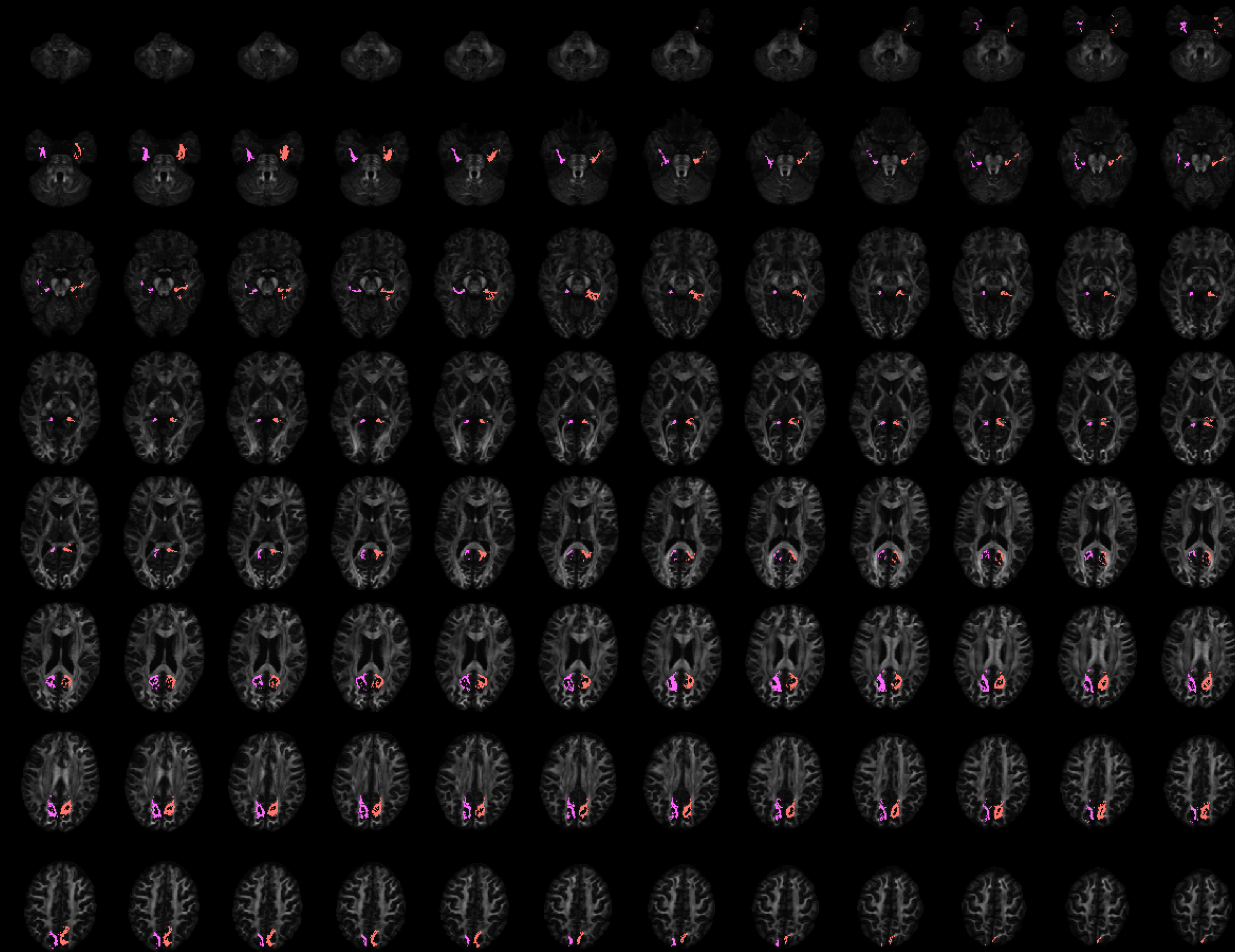

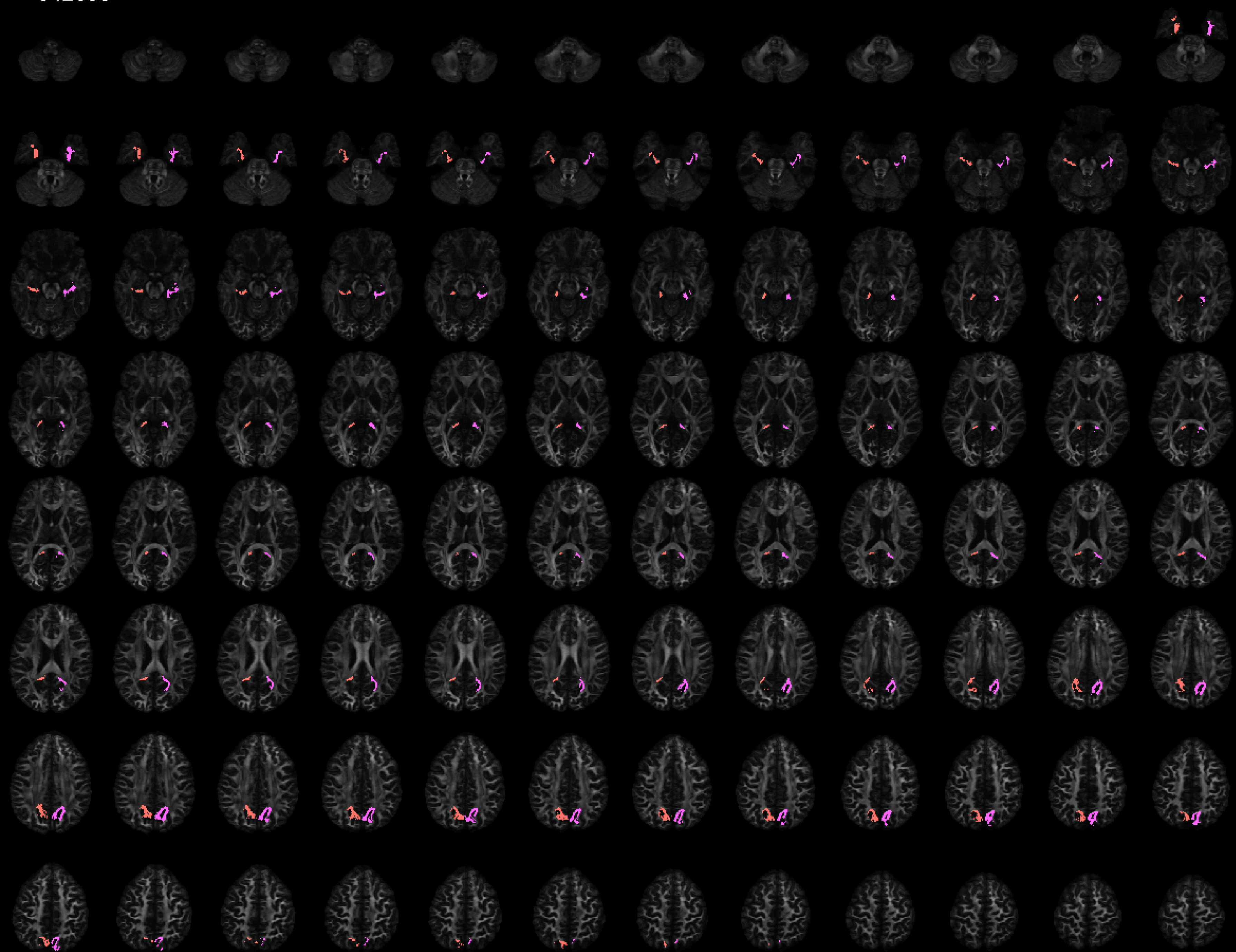

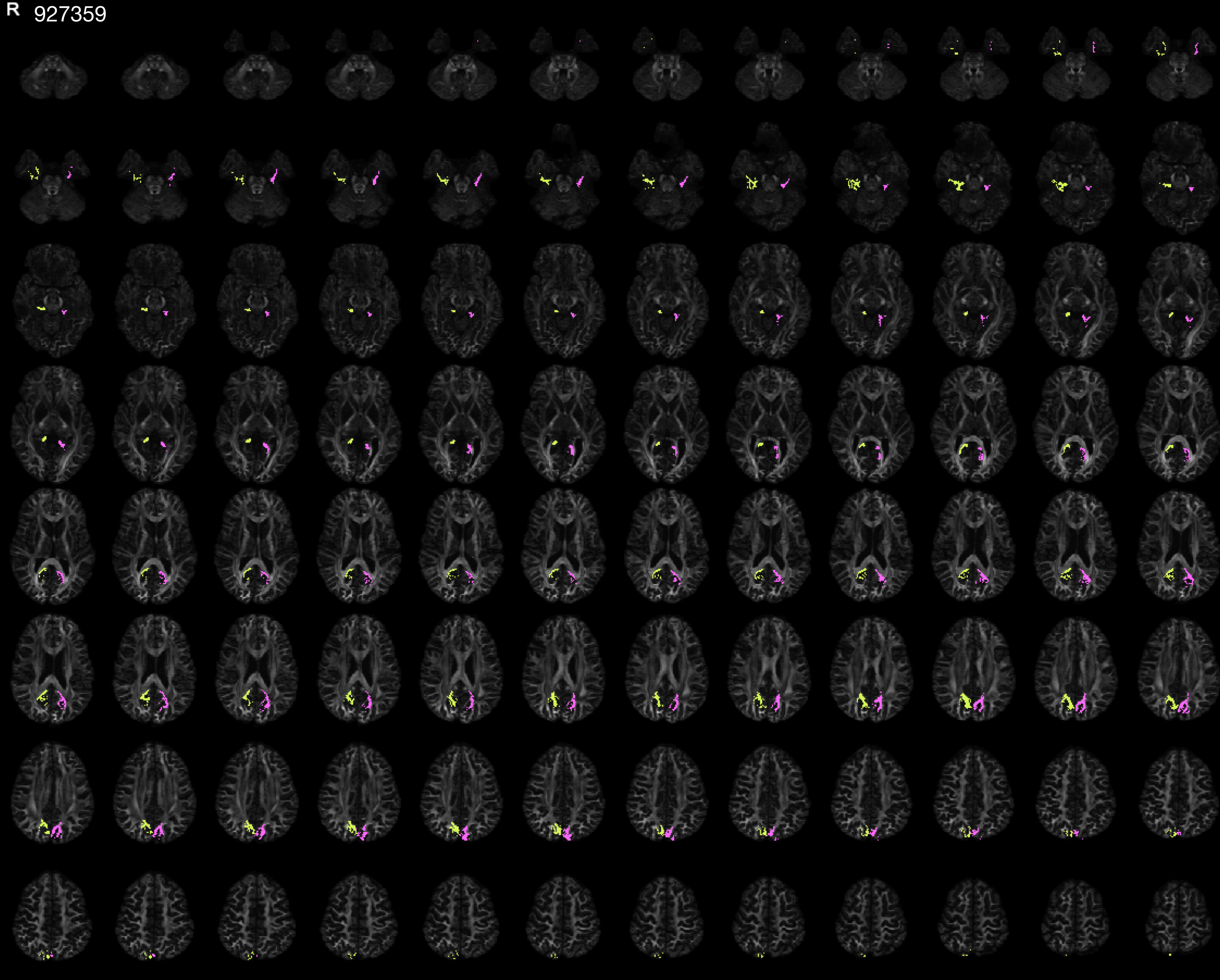

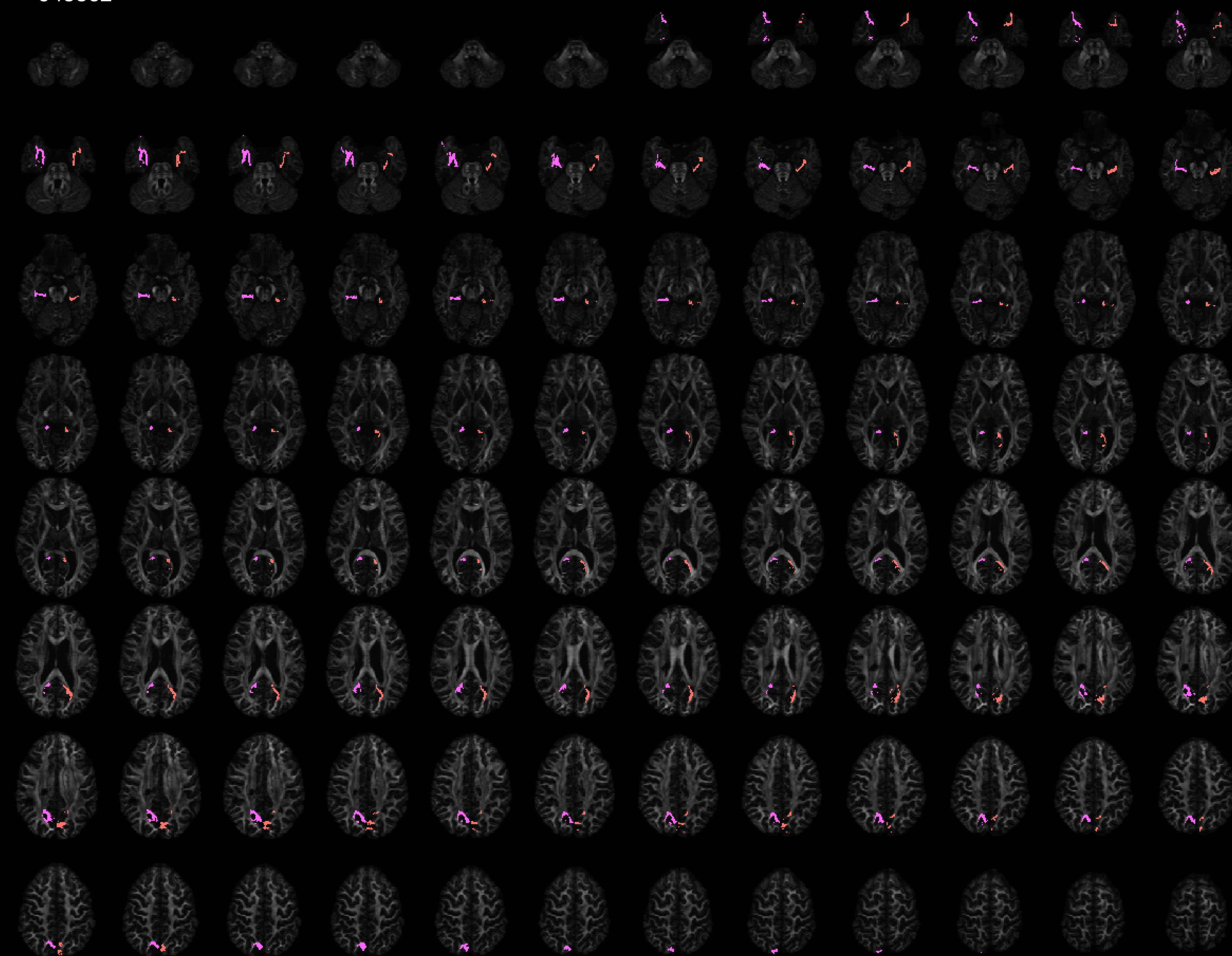

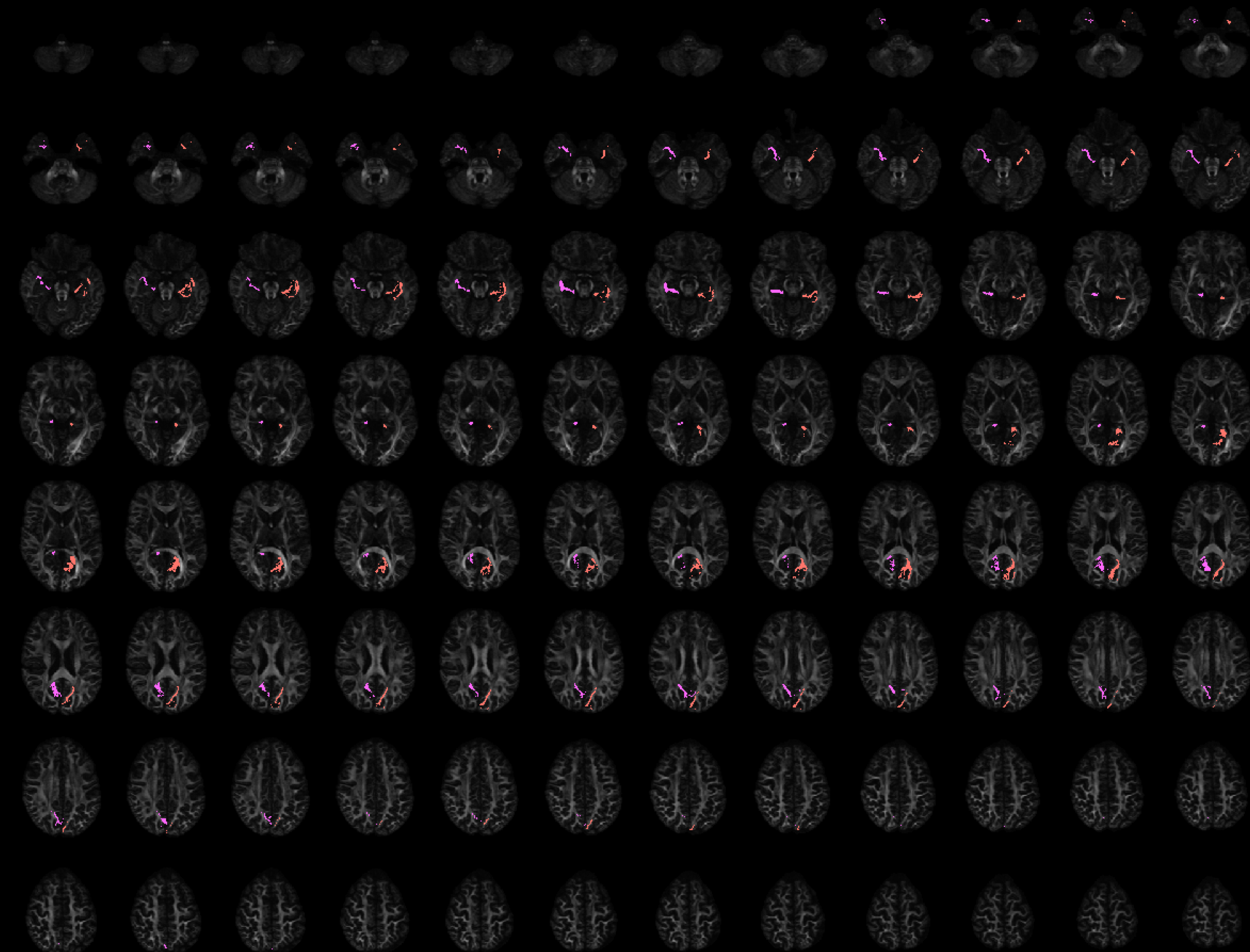

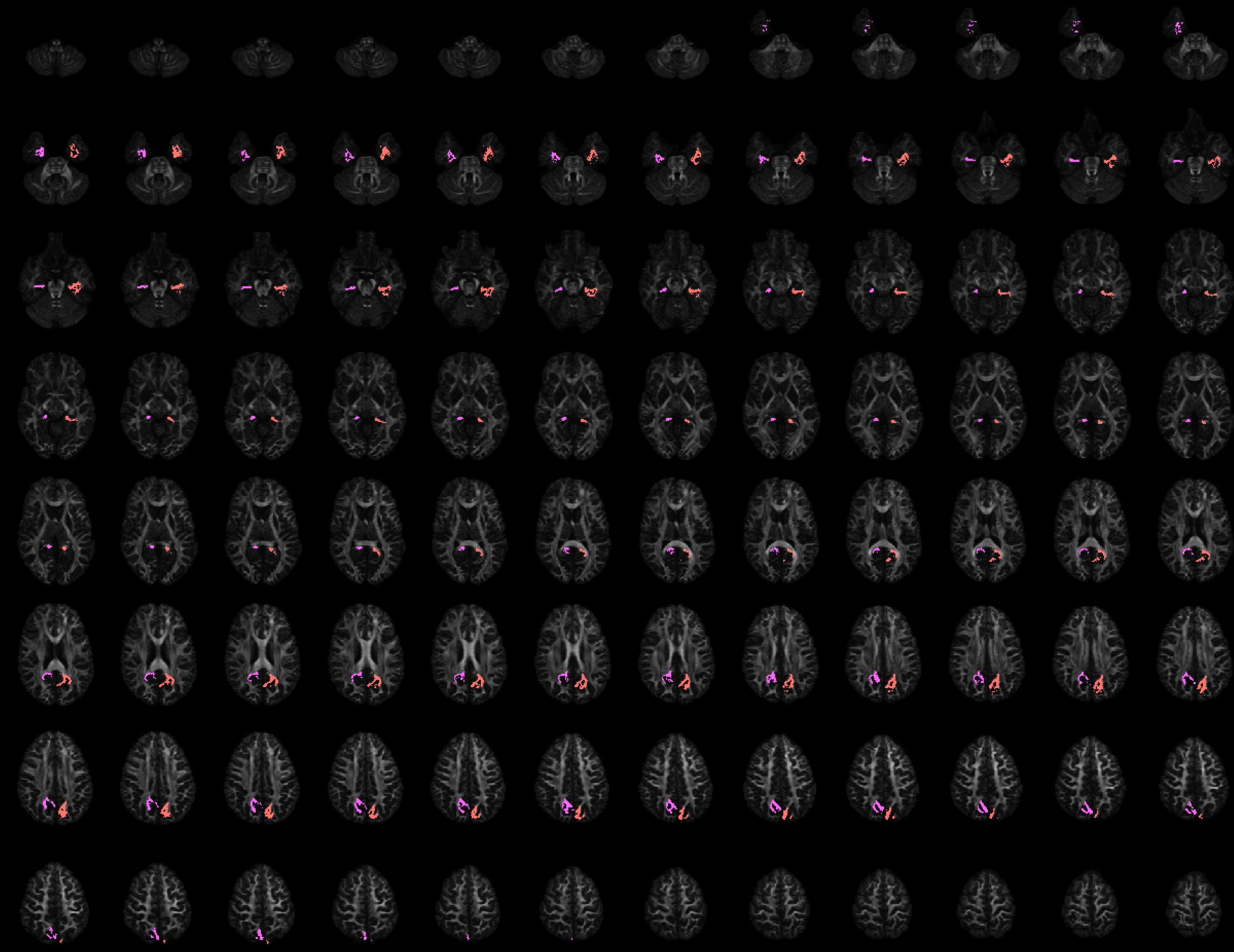

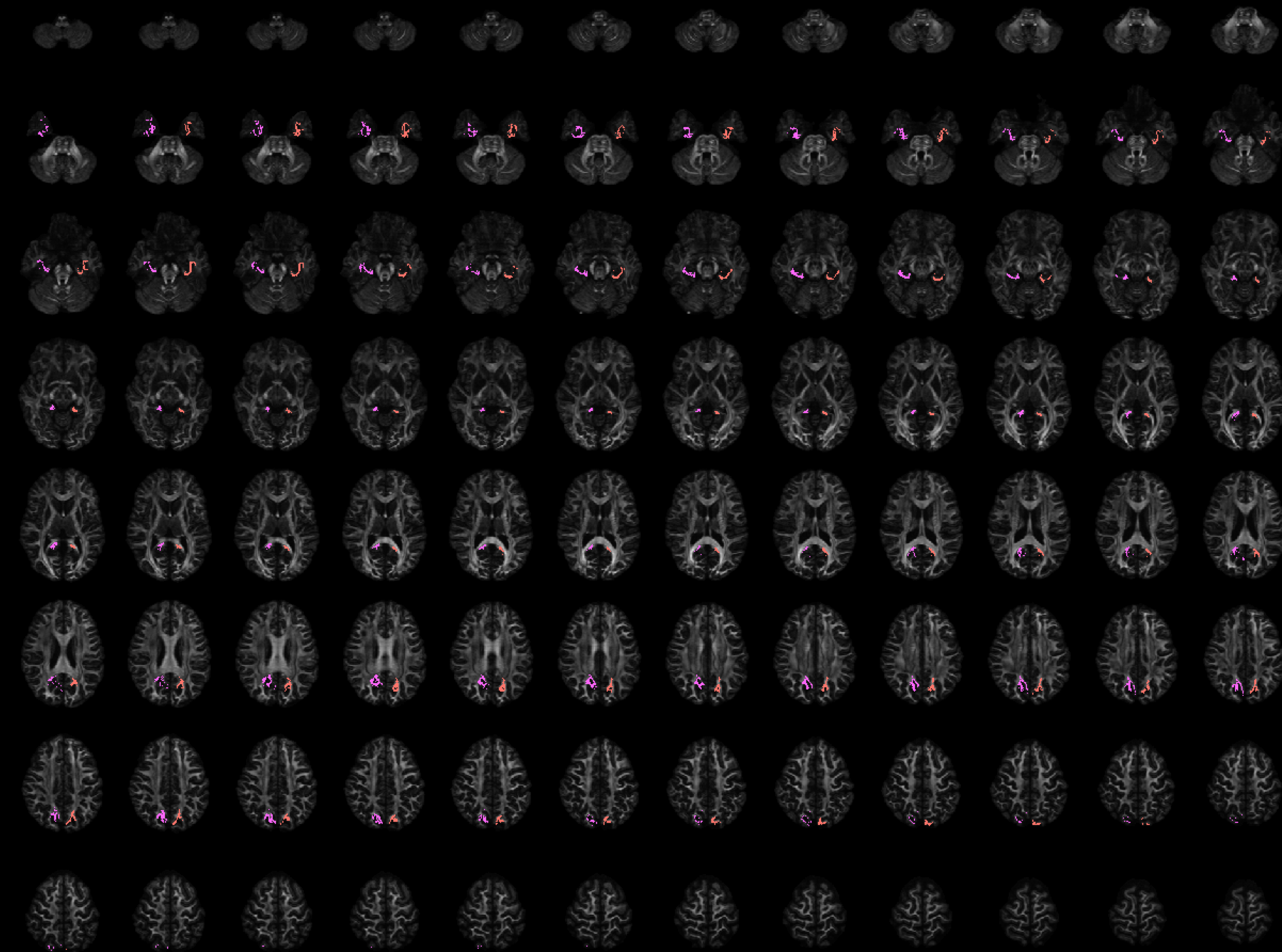

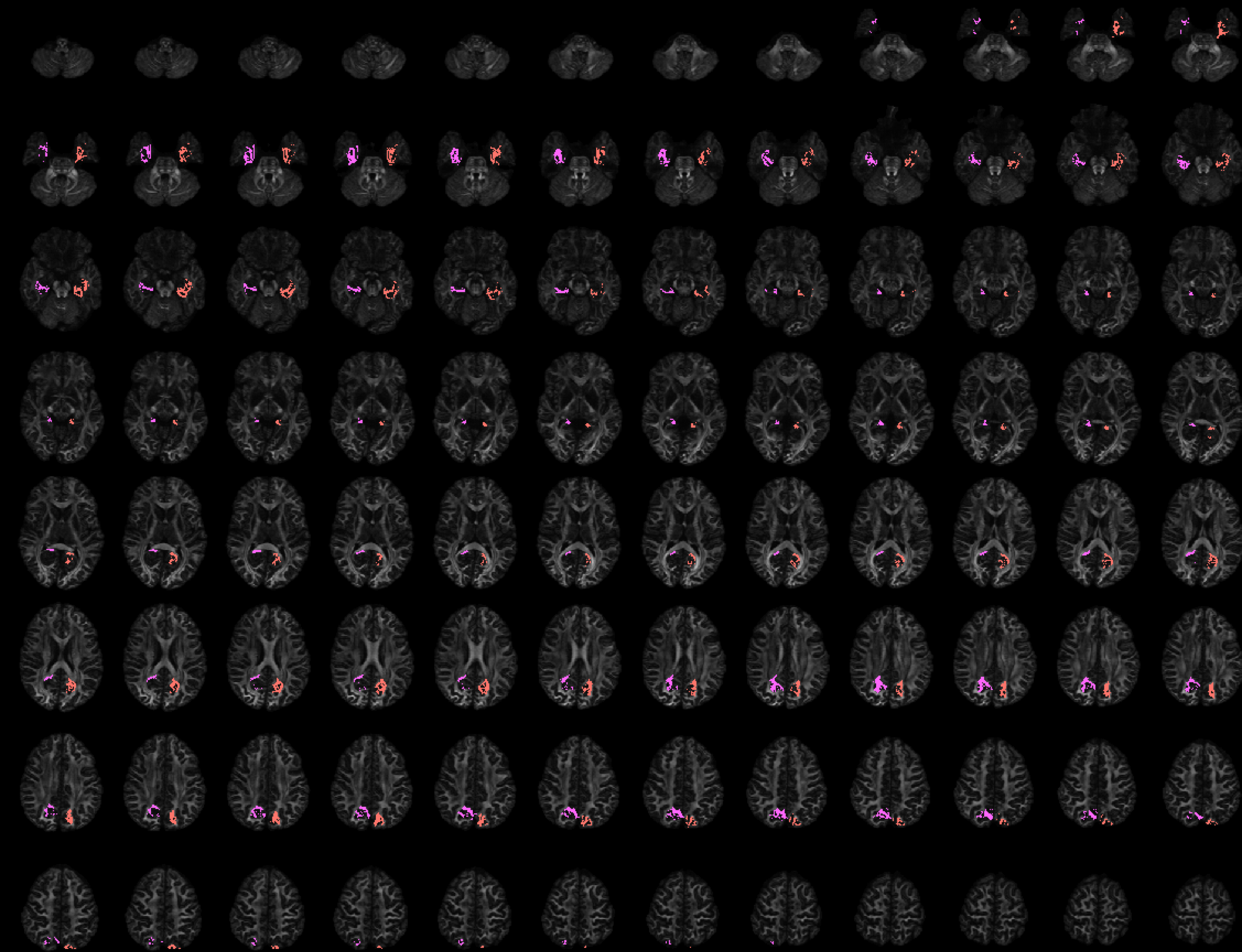

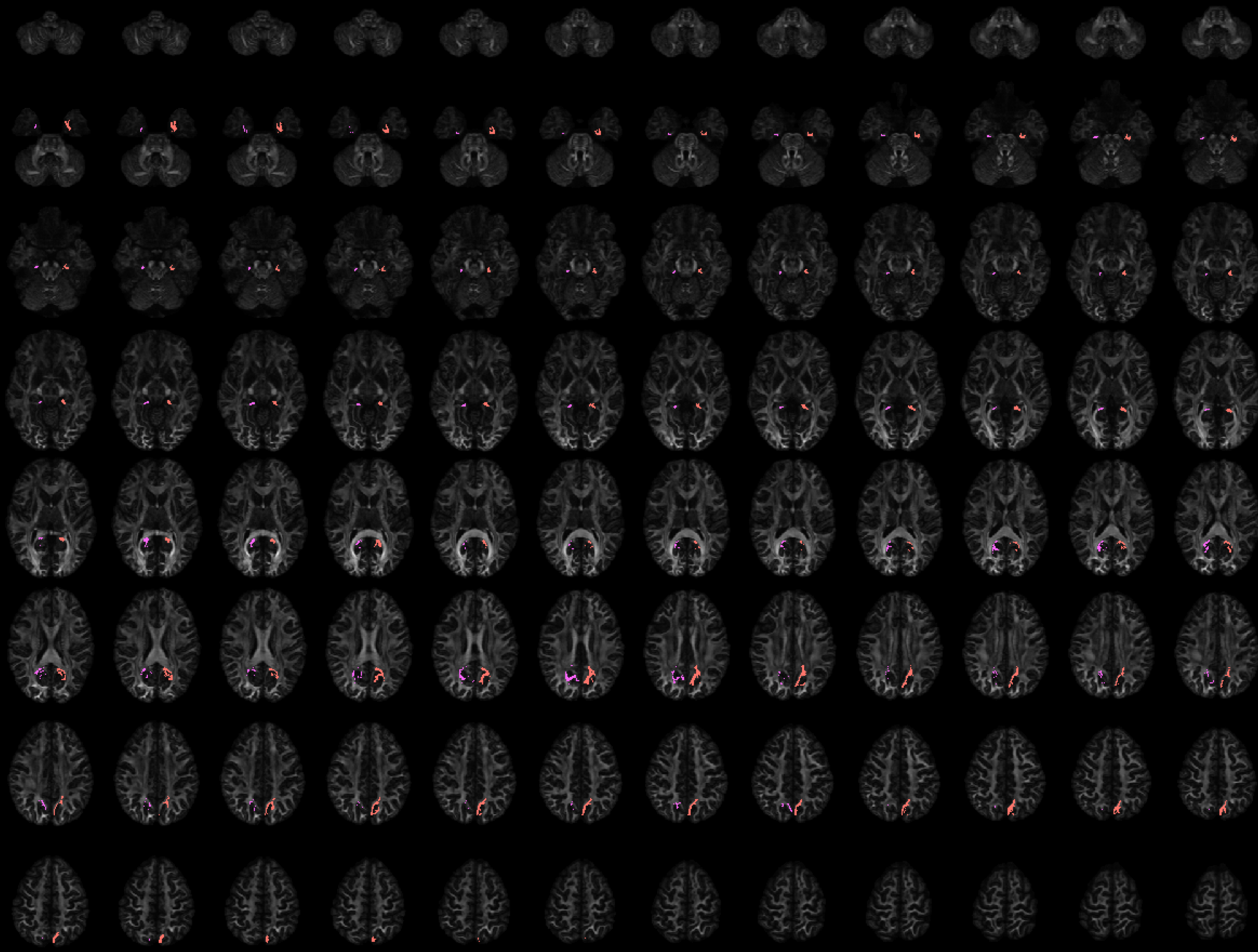

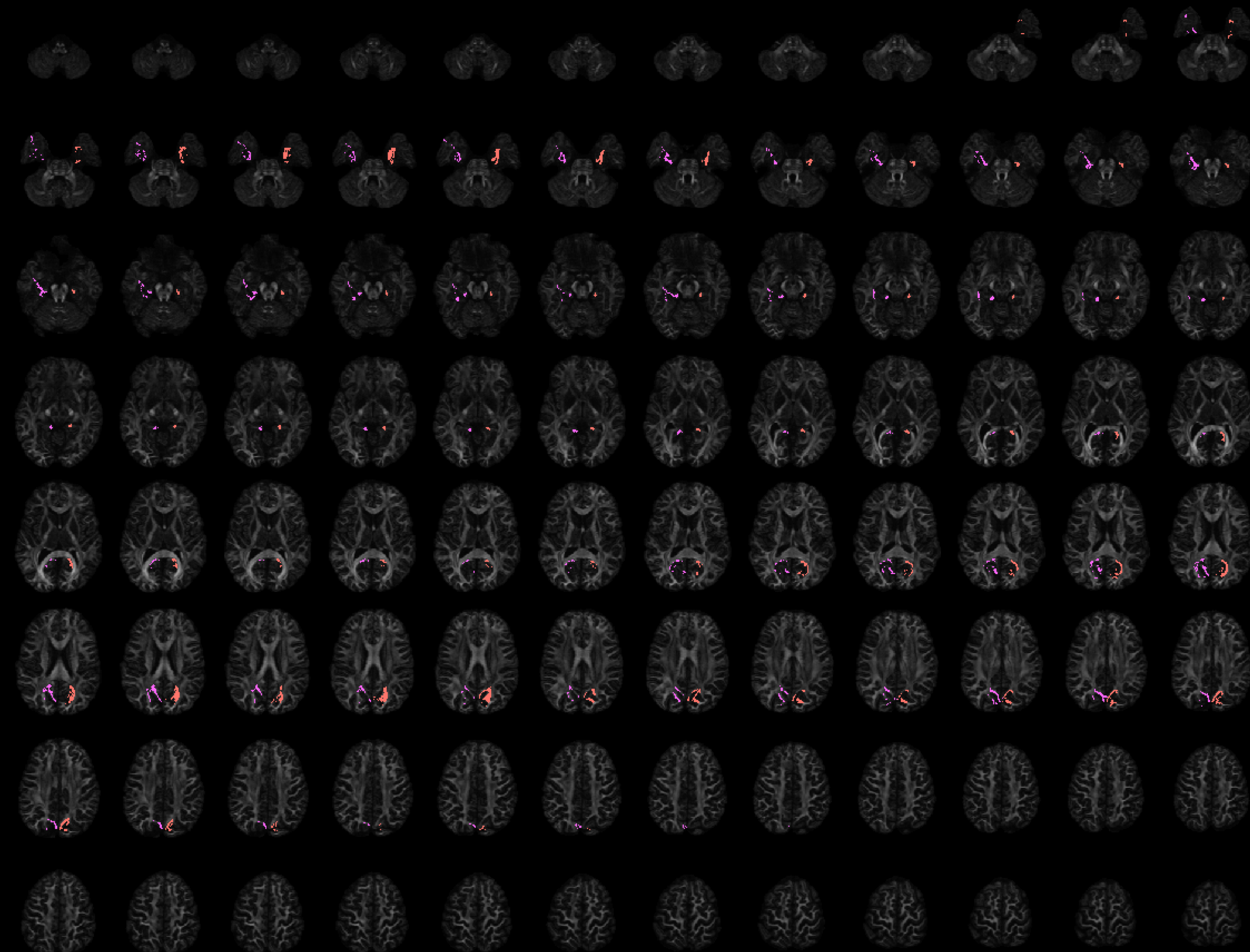

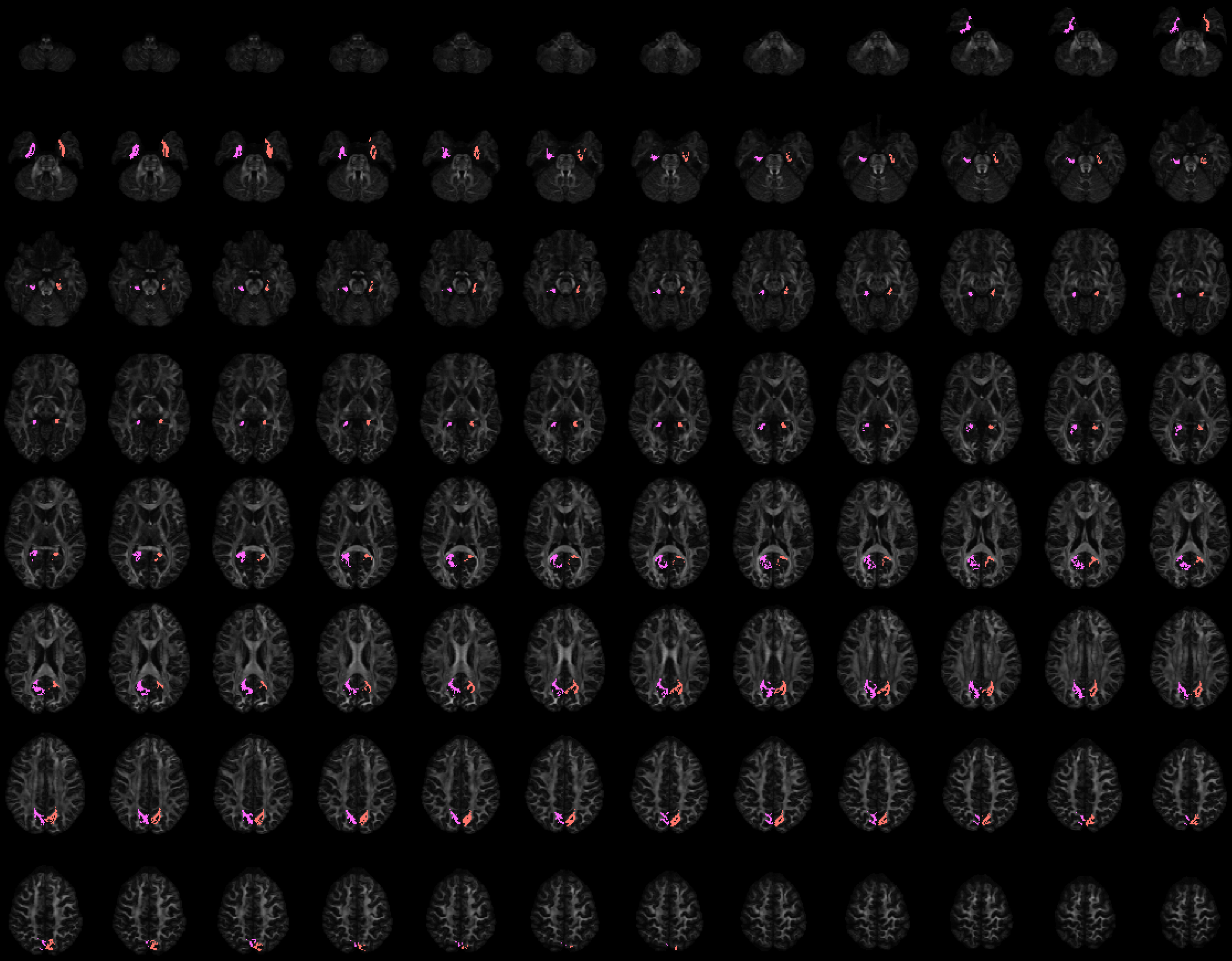

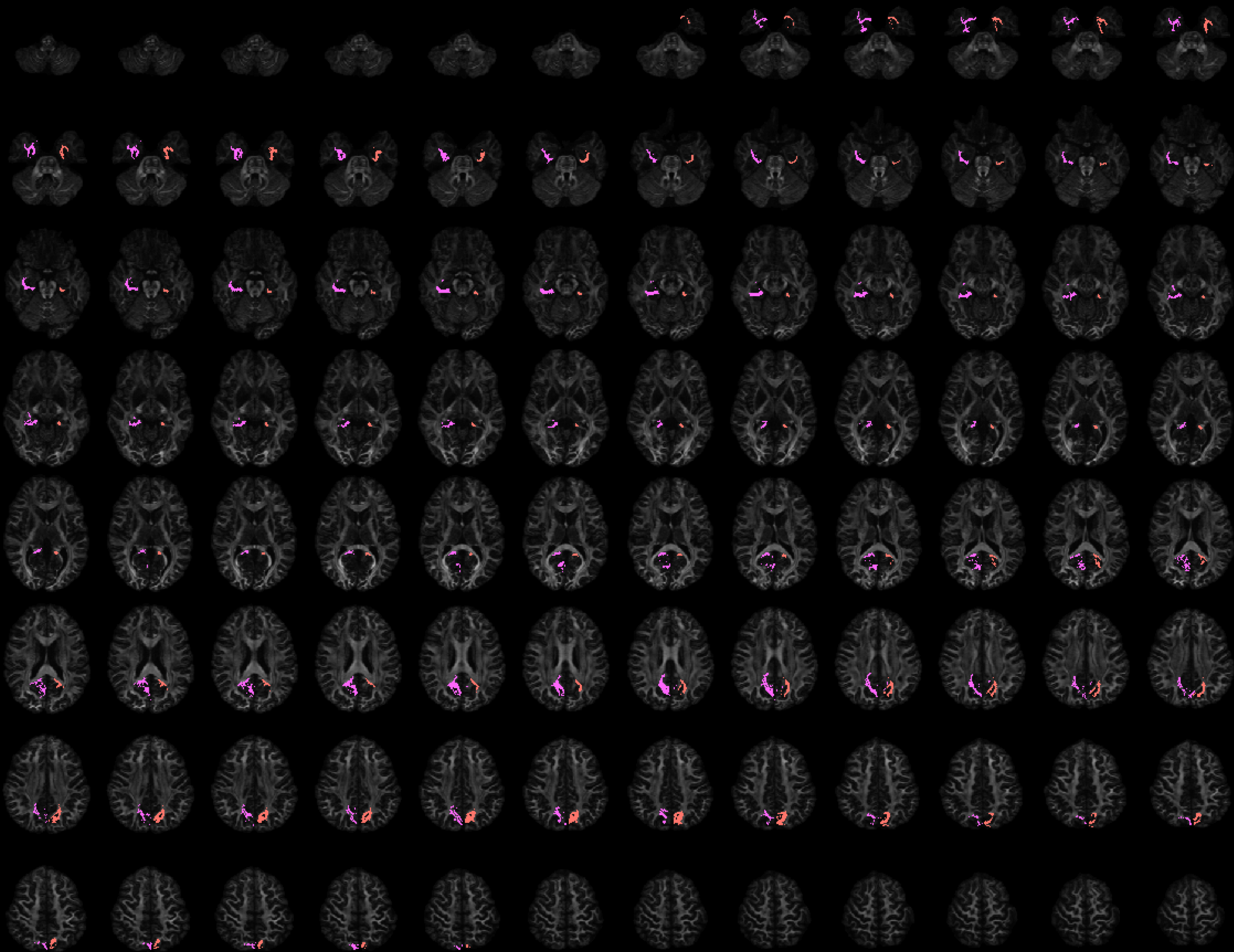

R 970764

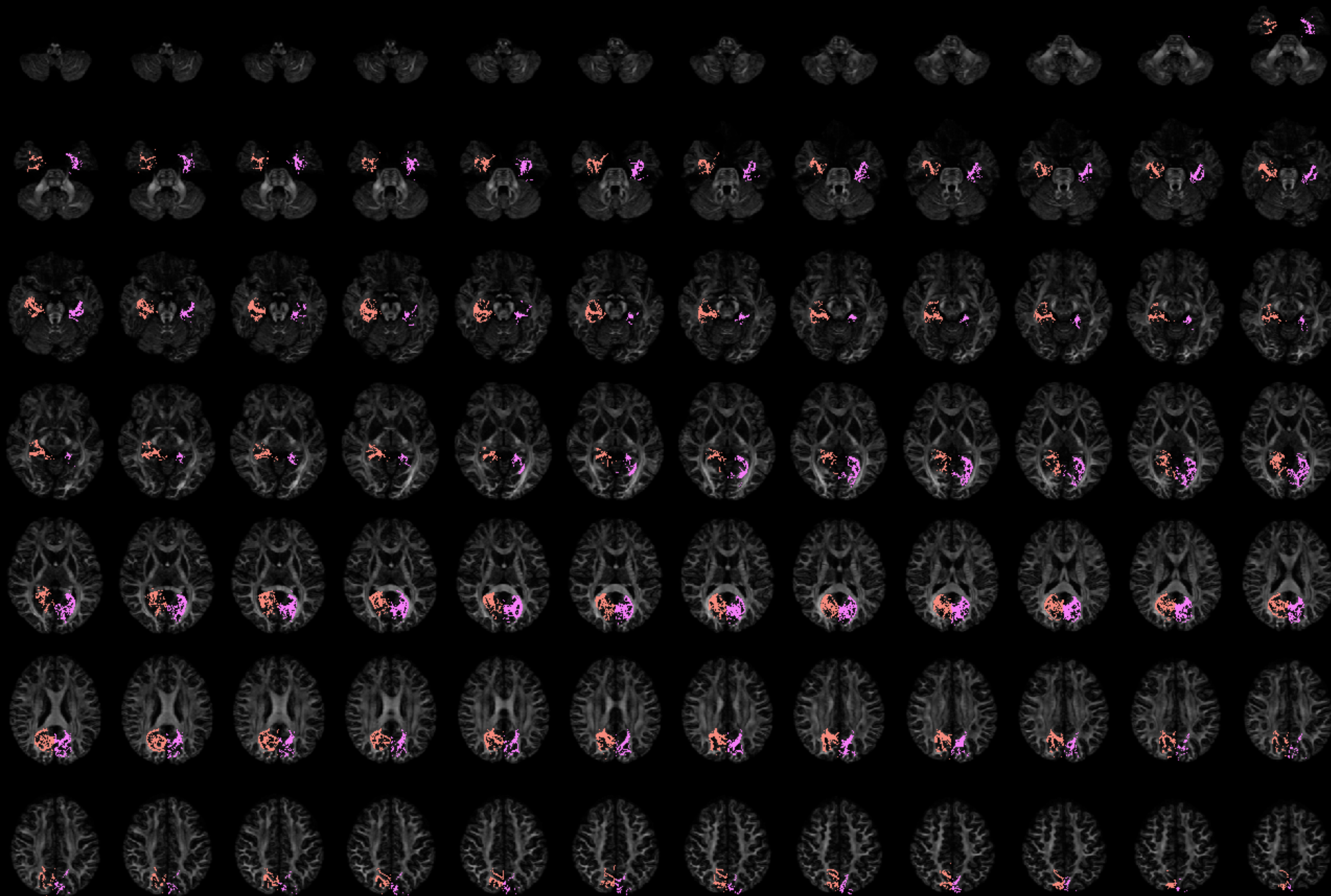

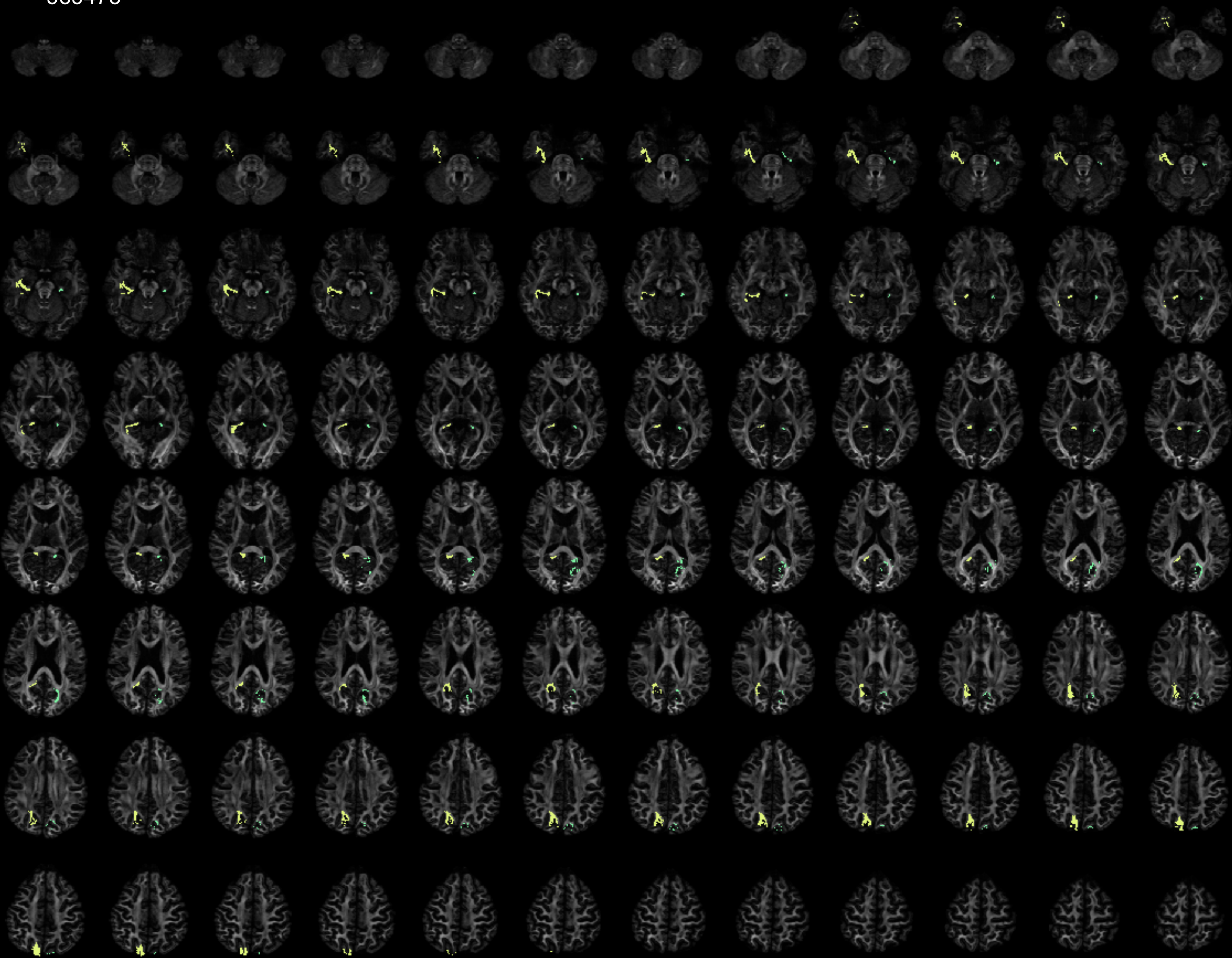

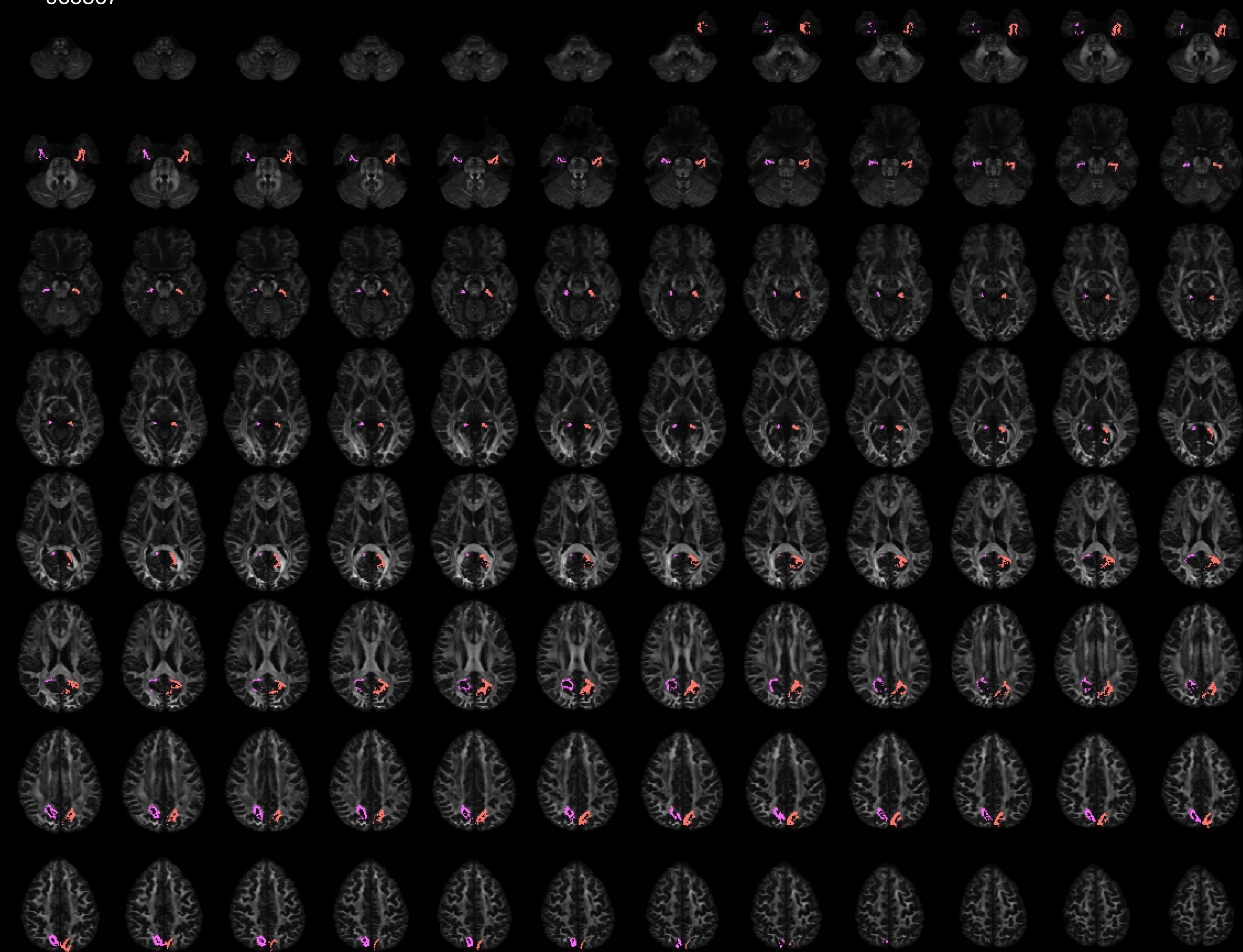

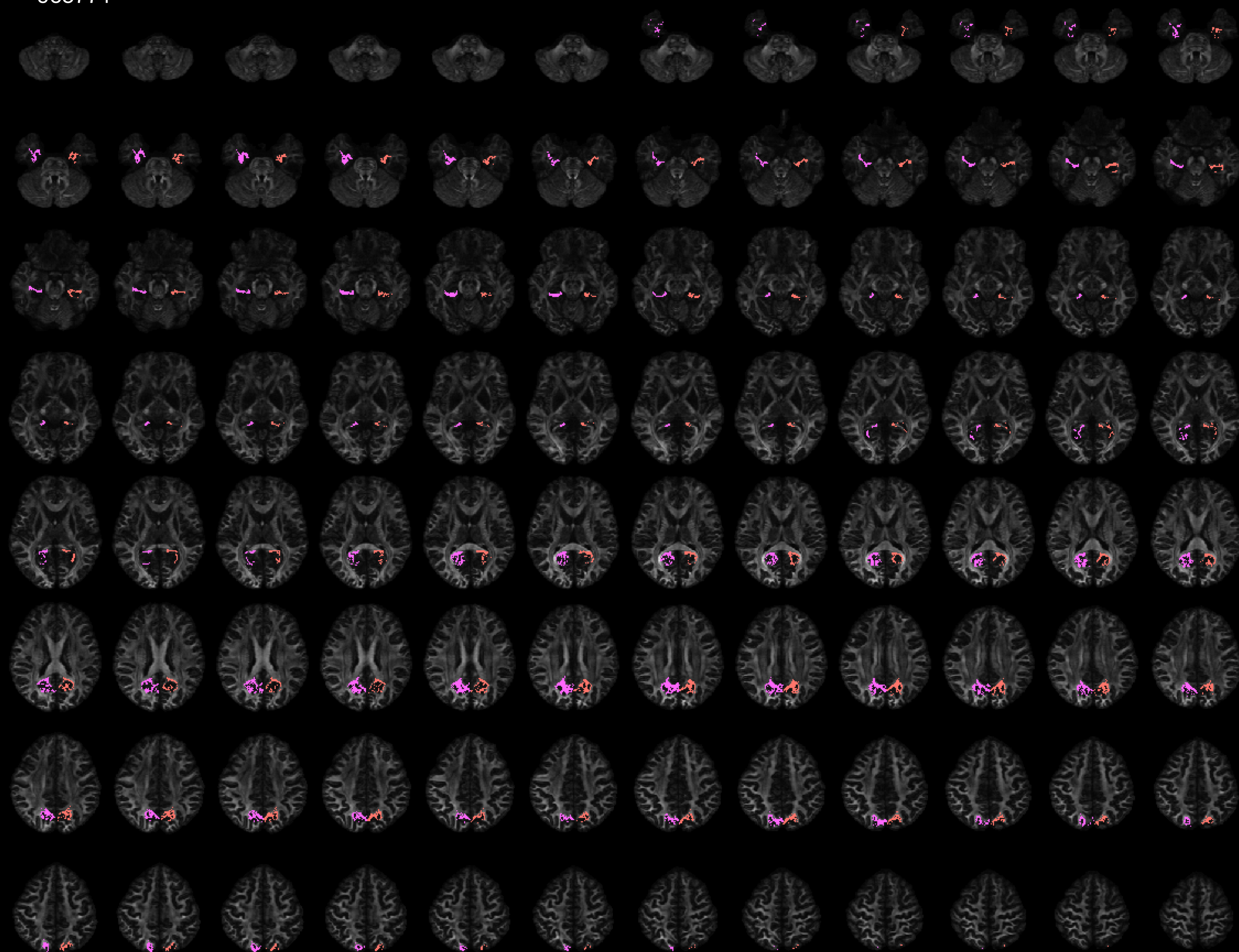

R 966975

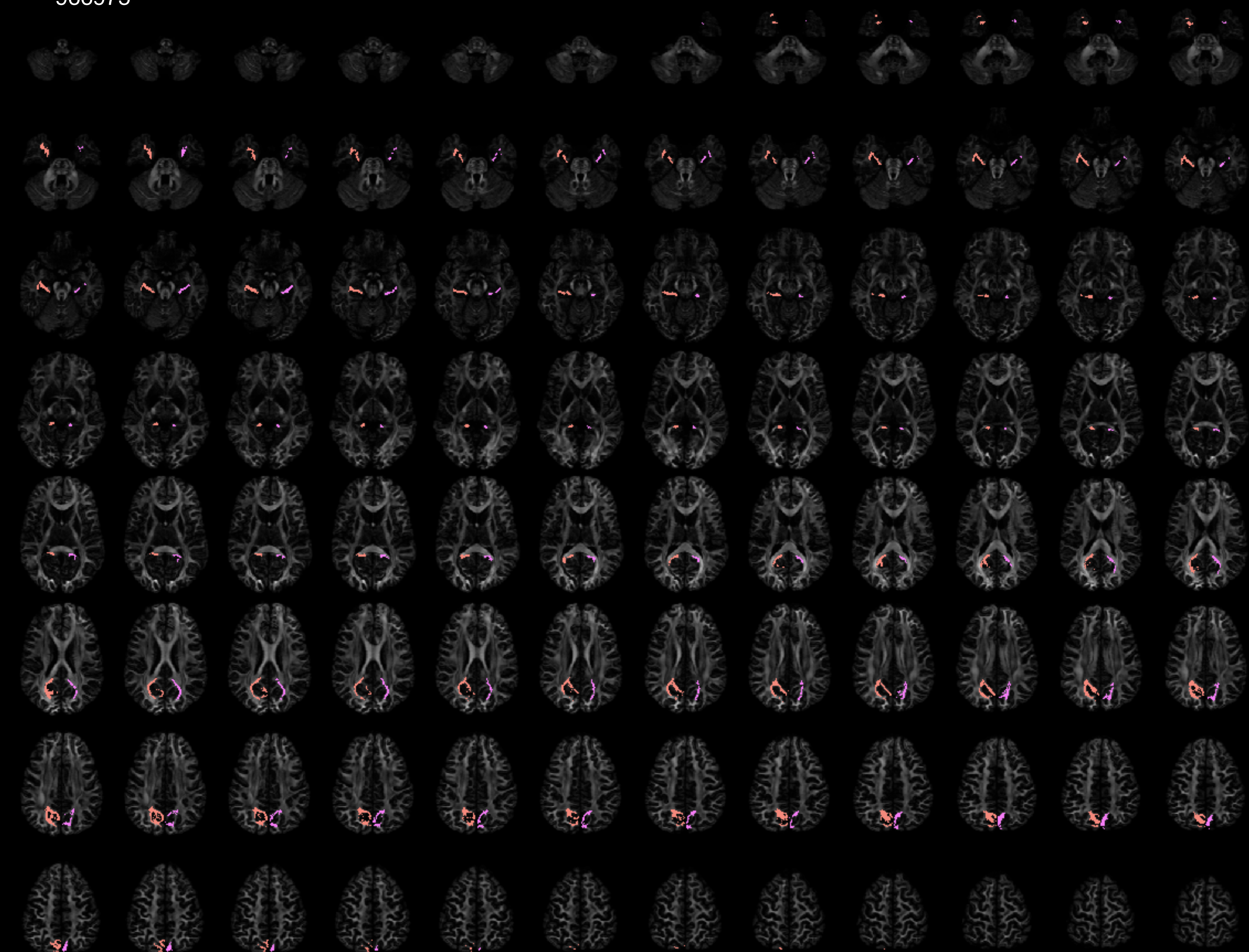

Supplement: Supplementary file 1 — Data S1: Supporting Information. [file HBM-45-e26771-s001.pdf]
